# A conserved ZFX/WNT3 axis modulates the growth and imatinib response of chronic myeloid leukemia stem/progenitor cells

**DOI:** 10.1186/s11658-023-00496-z

**Published:** 2023-10-20

**Authors:** Xiuyan Zhang, Yu Wang, Jinchang Lu, Lun Xiao, Hui Chen, Quanxue Li, Yuan-Yuan Li, Peng Xu, Changgeng Ruan, Haixia Zhou, Yun Zhao

**Affiliations:** 1 Cyrus Tang Medical Institute, Soochow University, Suzhou, 215123 China; 2 Jiangsu Institute of Hematology, NHC Key Laboratory of Thrombosis and Hemostasis, The First Affiliated Hospital of Soochow University, Suzhou, 215006 China; 3 Department of Vascular Surgery, The Affiliated Drum Tower Hospital, Nanjing University Medical School, Nanjing, 210008 China; 4 Shanghai-MOST Key Laboratory of Health and Disease Genomics, Shanghai Institute for Biomedical and Pharmaceutical Technologies, Shanghai, 200237 China; 5 National Clinical Research Center for Hematologic Diseases, Suzhou, 215006 China; 6 Collaborative Innovation Center of Hematology, Soochow University, Suzhou, 215006 China; 7 MOE Engineering Center of Hematological Disease, Soochow University, Suzhou, 21513 China

**Keywords:** ZFX, WNT3, CD34^+^ cells, Chronic myeloid leukemia, Imatinib mesylate

## Abstract

**Background:**

Zinc finger protein X-linked (ZFX) has been shown to promote the growth of tumor cells, including leukemic cells. However, the role of ZFX in the growth and drug response of chronic myeloid leukemia (CML) stem/progenitor cells remains unclear.

**Methods:**

Real-time quantitative PCR (RT–qPCR) and immunofluorescence were used to analyze the expression of ZFX and WNT3 in CML CD34^+^ cells compared with normal control cells. Short hairpin RNAs (shRNAs) and clustered regularly interspaced short palindromic repeats/dead CRISPR-associated protein 9 (CRISPR/dCas9) technologies were used to study the role of ZFX in growth and drug response of CML cells. Microarray data were generated to compare ZFX-silenced CML CD34^+^ cells with their controls. Chromatin immunoprecipitation (ChIP) and luciferase reporter assays were performed to study the molecular mechanisms of ZFX to regulate WNT3 expression. RT–qPCR and western blotting were used to study the effect of ZFX on β-catenin signaling.

**Results:**

We showed that ZFX expression was significantly higher in CML CD34^+^ cells than in control cells. Overexpression and gene silencing experiments indicated that ZFX promoted the in vitro growth of CML cells, conferred imatinib mesylate (IM) resistance to these cells, and enhanced BCR/ABL-induced malignant transformation. Microarray data and subsequent validation revealed that *WNT3* transcription was conservatively regulated by ZFX. WNT3 was highly expressed in CML CD34^+^ cells, and WNT3 regulated the growth and IM response of these cells similarly to ZFX. Moreover, WNT3 overexpression partially rescued ZFX silencing-induced growth inhibition and IM hypersensitivity. ZFX silencing decreased WNT3/β-catenin signaling, including c-MYC and CCND1 expression.

**Conclusion:**

The present study identified a novel ZFX/WNT3 axis that modulates the growth and IM response of CML stem/progenitor cells.

**Supplementary Information:**

The online version contains supplementary material available at 10.1186/s11658-023-00496-z.

## Background

Zinc finger protein X-linked (ZFX) is a zinc finger protein that is highly conserved in vertebrates. ZFX has both a C2H2-type zinc finger domain and an acidic activation domain, and acts as a transcriptional activator [1, 2]. At the same time, ZFX modulates chromatin architecture as a repressor of both core and linker histones [3, 4]. Several studies have demonstrated that ZFX controls the self-renewal of embryonic stem cells, hematopoietic stem cells, and peripheral T cells [5–7]. Transgenic mouse studies indicate that ZFX facilitates tumorigenesis [8], which is further supported by studies on various human tumors [9–24], including leukemia [16–18]. Overall, ZFX upregulation is a poor prognostic marker [20, 23], and ZFX silencing inhibits the growth of tumor cells and sensitizes them to chemotherapy [9–12, 15–19, 21]. The key to understanding how ZFX regulates tumor cells is to identify the direct transcriptional targets of ZFX. However, knowledge is limited [8, 10, 16, 21]. To date, only *DIS3L*, *c-Myc*, *Ptpmt1*, and *Idh2* are reported targets of ZFX [8, 10, 16].

Two independent reports have shown that ZFX promotes the growth of human chronic myeloid leukemia (CML) cells and confers imatinib mesylate (IM) resistance to these cells [17, 18]. CML is a hematological malignancy originating from normal hematopoietic stem cells, and leukemic stem cells play a critical role in disease pathology [25, 26]. However, the role of ZFX in CML stem/progenitor cells has not yet been elucidated.

The Wnt/β-catenin pathway consists of multiple WNT ligands, frizzled (FZD) receptors, and signaling intermediates [27], playing a critical role in multiple cancers [28–31], including CML [31–43]. For instance, β-catenin-deficient mice constrain BCR/ABL^+^ leukemic stem cells, suggesting a pivotal role of this pathway in disease initiation [32, 33]. Meanwhile, BCR/ABL directly phosphorylates β-catenin and stabilizes it [34]. The activation of this pathway also contributes to disease progression and chemotherapy resistance [35–39]. In line with this, recent reports have shown that β-catenin is important for GAS6/AXL or PRMT5 inhibition-induced growth suppression of CML stem/progenitor cells [40, 41]. Additionally, WNT/β-catenin signaling plays an important role in mesenchymal stem cell (MSC)-mediated protection of CML stem/progenitor cells from tyrosine kinase inhibitor (TKI) treatment, partially through the interaction of MSC-secreted WNT ligand and FZD receptor on the surface of leukemic stem/progenitor cells [42]. Several FZD receptors are highly expressed in CML CD34^+^ cells, and FZD4 silencing inhibits the growth of these cells and sensitizes them to TKI treatment, even in the absence of MSCs, implying that CML CD34^+^ cells may secrete WNT ligands [43]. Moreover, the inhibitor against *O*-acyl transferase porcupine (PORCN) (WNT974) in combination with TKI significantly reduced the growth of CML stem/progenitor cells in immunodeficient mice compared with TKI alone [43]. However, the expression of WNT ligand in CML stem/progenitor cells has not been investigated, and whether ZFX is implicated in WNT/β-catenin signaling activation in CML stem/progenitor cells is still unknown.

To address the above questions, ZFX expression in CML stem/progenitor cells was measured, and the effects of ZFX silencing on the growth and IM response of these cells were studied. In addition, transcriptome data were generated to delineate the mechanism of how ZFX regulates CML stem/progenitor cells. Our data have revealed a conserved ZFX/WNT3 regulatory axis, which modulates the growth and IM response of BCR/ABL^+^ cells partially via elevated WNT3 secretion and the activation of WNT/β-catenin signaling. The present study deepens our understanding of the molecular pathology of CML and possibly benefits disease management.

## Materials and methods

### Cells and human samples

K562 cells (no. SCSP-5054) and 293 T cells (no. SCSP-502) were obtained from the Cell Bank of the Chinese Academy (www.cellbank.org.cn). K562 cells were maintained in Rosewell Park Memorial Institute (RPMI) 1640 medium supplemented with 10% fetal bovine serum (FBS), and 293 T cells were maintained in Dulbecco’s modified Eagle medium (DMEM) plus 10% FBS. Murine BaF3 cells (from Dr. Connie J. Eaves, Terry Fox Laboratory, the British Columbia Cancer Research Institute) were maintained in RPMI 1640 medium supplemented with 10% FBS and 5 ng/mL mIL-3. Murine BaF3-BCR/ABL cells were generated by *BCR/ABL* lentiviral transduction and were maintained in RPMI 1640 medium plus 10% FBS. Bone marrow cells (BMCs) from human CML patients and healthy donors were obtained from the Hematological Biobank, Jiangsu Biobank of Clinical Resources. Nucleated cells (unfractioned BMCs) were obtained using a gradient centrifuge with lympholyte-H cell separation media (Cedarlane Laboratories, Burlington, NC, USA), and CD34^+^ cells were purified using an EasySep CD34 positive selection kit (STEMCELL Technologies, Vancouver, BC, Canada). The clinical characteristics of CML patients recruited in the present study are summarized in Additional file 1: Table S1.

### Lentivirus production and transduction

The lentiviral vectors for gene silencing were purchased from GenePharma Co., Ltd. (Shanghai, China), and the sequences of these short hairpin RNAs (shRNAs) are presented in Additional file 1: Table S2. The cDNA of WNT3 was amplified by RT–PCR and subcloned into a lentiviral vector [44]. The primer sequences to amplify WNT3 are listed in Additional file 1: Table S3. Lentivirus was produced as previously described [44].

Normal bone marrow (NBM) and CML CD34^+^ cells were transduced with concentrated lentivirus, and the transduced CD34^+^ cells were isolated using fluorescence-activated cell sorting (FACS) (BD FACSAria III, Becton Dickinson, Franklin Lakes, NJ, USA). A total of 1000 FACS-isolated cells were plated in methylcellulose medium (MethoCult H4230, STEMCELL Technologies) for the colony-forming cell (CFC) assay, supplemented with a cocktail of cytokines, including stem cell factor (SCF, 50 ng/mL), interleukin-3 (IL-3, 20 ng/mL), interleukin-6 (IL-6, 20 ng/mL), granulocyte macrophage colony-stimulating factor (GM-CSF, 20 ng/mL), granulocyte colony-stimulating factor (G-CSF, 20 ng/mL), and erythropoietin (EPO, 3 IU/mL). The colonies were classified and counted 14–16 days later. For CFC assays with imatinib mesylate (IM), SCF and EPO were not supplemented.

### RNA extraction and RT–qPCR

Total RNA was extracted using an RNAprep Pure Micro kit (Tiangen, Beijing, China) and reverse transcribed into cDNA with a RevertAid First Strand cDNA Synthesis Kit (Thermo Scientific, Waltham, MA, USA). RT–qPCR was performed using SYBR Green PCR MasterMix with a Prism® 7500 real-time PCR system (ThermoFisher, Applied Biosystems, Foster City, CA, USA). The expression of each transcript was normalized to that of *β-ACTIN*. To compare the expression of individual transcripts in different samples, the expression in the test group was normalized to that in the control group and is shown as relative expression. The sequences of gene-specific primers are summarized in Additional file 1: Table S3.

### Western blotting

Protein samples were prepared using protein lysate buffer (Beyotime, Shanghai, China) supplemented with phenylmethanesulfonyl fluoride (PMSF, final concentration 1 mM). Equal amounts of protein samples were separated with sodium dodecyl sulfate–polyacrylamide gel electrophoresis (SDS–PAGE) and transferred from the electrophoresed gel onto a polyvinylidene difluoride (PVDF) membrane (Millipore, Billerica, MA, USA). To collect secreted protein samples, same amounts of cells were cultured with serum-free medium for 24 h, and the culture medium was processed with Amicon Ultra Centrifugal Filter (UFC801096, Millipore) to concentrate secretion proteins, and the remaining samples were analyzed by western blotting. Ponceau S staining was used as a loading control. The information of antibodies used in this study is listed in Additional file 1: Table S4. The specificity of WNT3 antibody was verified by gene silencing and forced expression experiments. The film was developed by a Kodak Medical X-ray Processor 102 (Kodak, Rochester, NY, USA) using the ECL detection system (GE Healthcare Life Sciences, Piscataway, NJ, USA).

### Immunofluorescence

Immunofluorescence analysis was performed as previously described [18]. In brief, each sample (1 × 10^5^ cells) was transferred, air-dried, fixed on coated slides (Thermo Scientific), and incubated with primary antibody (anti-ZFX, PAB20245, 1/100; Abnova) overnight at 4 °C in a humidified container. The sample was incubated with an appropriate secondary antibody and covered with Prolong Gold Antifade reagent (Life Technologies, Grand Island, USA). Finally, images were obtained and analyzed with a confocal microscope (FV1000MPE-share; Olympus, Tokyo, Japan).

### Intracellular flow cytometry

After washing with cold PBS, cells of each sample (1 × 10^6^ cells) were fixed in 4% formaldehyde for 15 min at room temperature, and permeabilized in 90% methanol for a minimum of 10 min on ice. All the samples were washed in PBS to remove methanol, and then resuspended in diluted activated β-catenin antibody (CST, #8814) or concentration-matched isotype control IgG (CST, #3900) at 4 ℃ overnight. The cells were incubated with fluorochrome-conjugated secondary antibody for 1 h at room temperature. Finally, the samples were resuspended with PBS and analyzed on a flow cytometer (Calibur, BD).

### CRISPR/dCas9-mediated overexpression

To activate the expression of Zfx in BaF3 or BaF3–BCR/ABL cells, the CRISPR synergistic activation mediator (SAM) system was utilized. These cells were transduced with dCas9–VP64–blast (Addgene #61425) and MS2–P65–HSF1 activator complex with a 2A hygromycin resistance marker (MPHv2) (Addgene #89308) and were selected by blasticidin (10 μg/mL) (Solarbio LIFE SCIENCES, Beijing, China) and hygromycin (300 μg/mL) (Solarbio LIFE SCIENCES) for 5 days. Finally, these cells were transduced with lenti-sgRNA (MS2) zeo (Addgene #61427) and selected by zeocin (200 μg/mL) (Solarbio LIFE SCIENCES) for 7 days. The sequences of sgRNAs used in this study are listed in Additional file 1: Table S5. The drug-resistant cells were collected, and the expression of ZFX was analyzed by RT–qPCR and Western blotting.

### Animals

The experimental procedure to generate a mouse model of BCR/ABL^+^ leukemia was as previously described [45]. In brief, 6–8-week-old female BalB/C mice were lethally irradiated and randomly allocated to test and control groups. The test and control cells were mixed with 5.0 × 10^5^ mouse bone marrow cells, and the mixture was injected intravenously into the irradiated mice. These mice were observed carefully for signs of weight loss or lethargy. When the diseased mice were near death, they were dissected, and the weights of the spleens and livers were measured. Cells from the spleen, liver, bone marrow, and peripheral blood were analyzed by flow cytometry, RT–qPCR, and western blotting.

### Microarray analysis

Three pairs of ZFX-silenced and control CML CD34^+^ cells were harvested for microarray analysis using Agilent whole human genome oligo-chips (4 × 180 K) from Shanghai Biotechnology Corporation. Data were normalized with quantile normalization, and the differentially expressed transcripts were extracted with consistent criteria (*P* < 0.05 and fold change > 2).

### Dual luciferase reporter assay

The promoter region of *WNT3* was subcloned into a pGL3–Basic vector to generate pGL3–WNT3, and a series of *WNT3* mutant reporter vectors were constructed. The primers used in this part are summarized in Additional file 1: Table S3. Briefly, 3 μg reporter vector (containing firefly luciferase) and 0.3 μg internal control reporter vector (containing renilla luciferase) were delivered into cells by a Nucleofector device (Lonza, Basel, Switzerland) following the manufacturer’s instructions. Forty-eight hours later, equal amounts of cells were harvested, and reporter activities were assessed by a Luminoskan Ascent reader (Thermo Scientific) using the Dual-Luciferase Reporter Assay System (Promega, Madison, WI, USA). The reporter activity was normalized to the control reporter activity, and the relative transcription activities of individual samples were compared.

### Chromatin immunoprecipitation (ChIP) assay

ChIP was performed as previously described [46]. Briefly, 2 × 10^6^ cells were completely washed with PBS and fixed with formaldehyde. Cell lysate was treated with sonication to obtain DNA fragments (200–1000 bp). Then, it was incubated with ZFX antibody (PAB20245, Abnova), RNA polymerase II antibody, and immunoglobulin G isotype antibody. The enriched DNA fragments were then purified, quantified, and analyzed by conventional PCR or qPCR. The gene-specific primers used for this assay are summarized in Additional file 1: Table S3.

### Statistical analysis

All values are represented as the mean ± SEM from more than three biological replicates, and statistical analysis was performed with Student’s *t*-test, in which a *P* < 0.05 was considered significant. The Kaplan–Meier method was used to study the survival tendency, and the *P*-value was estimated using the log-rank test.

## Results

### Increased expression of ZFX modulates the growth and imatinib mesylate response of BCR/ABL^+^ leukemic cells

**Fig. 1:**
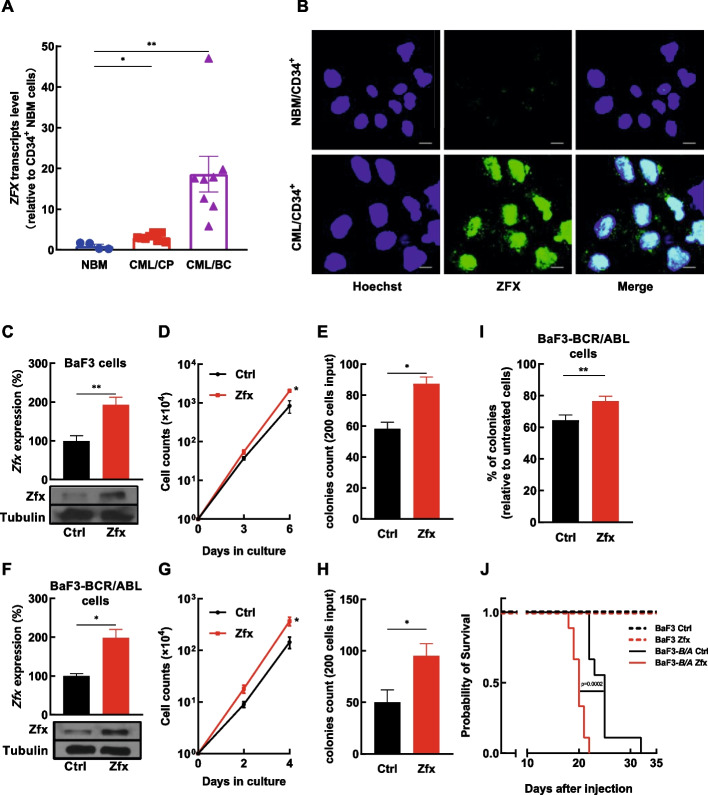
Highly expressed zinc finger protein X-linked enhances the transformation ability of BCR/ABL. **A** CD34^+^ cells were obtained from normal bone marrow (NBM) of healthy donors (*n* = 4), CML patients in chronic phase (CP, *n* = 8), and those in blast crisis (BC, *n* = 8). The expression of *zinc finger protein X-linked* (*ZFX*) in these cells was analyzed by RT–qPCR. **B** The expression of ZFX in CD34^+^ cells from NBM and a CML patient in chronic phase was analyzed by confocal microscopy, and the representative photos are shown (scale bar = 5 μm). **C** CRISPR/dCas9 technology was utilized to increase the expression of Zfx in mIL-3-dependent BaF3 cells, and the expression of Zfx was analyzed by RT–qPCR and western blotting. **D**, **E** The growth and colony-forming cell (CFC) production of Zfx-overexpressing and control BaF3 cells were measured in the presence of mIL-3 (5 ng/mL). **F** CRISPR/dCas9 technology was utilized to increase the expression of Zfx in BaF3-BCR/ABL cells, and the expression of Zfx was analyzed by RT–qPCR and western blotting. **G**, **H** The growth and CFC production of Zfx-overexpressing and control BaF3-BCR/ABL cells were analyzed (without mIL-3). **I** Zfx-overexpressing and control BaF3-BCR/ABL cells were subjected to CFC analysis with or without 2 μM imatinib mesylate (IM), and the percentage of CFC production relative to untreated cells was compared. **J** Zfx-overexpressing and control BaF3 cells and BaF3–BCR/ABL cells were injected into lethally irradiated mice, and the survival was analyzed with the Kaplan–Meier method (log-rank test, **** mean *P* < 0.0001). Data are presented as the mean ± SEM, and Student’s *t*-test was used to estimate the *P*-values (*P < 0.05 and **P < 0.01)

Previously, we reported that ZFX was upregulated in various leukemic cells and sustained the growth of these cells [18]. However, the role of ZFX in leukemia stem/progenitor cells has not been investigated. First, CD34^+^ cells were collected from the bone marrow of both healthy donors and CML patients, and the transcript expression of *ZFX* was analyzed by RT–qPCR and compared with *ZFX* expression in unfractioned BMCs reported previously [18]. The analysis showed that *ZFX* expression was significantly higher in CD34^+^ cells than in unfractioned BMCs (Additional file 1: Fig. S1A), which suggested that ZFX may play a crucial role in both normal and CML stem/progenitor cells. Next, *ZFX* expression in CD34^+^ cells from CML patients in both chronic phase (CP) and blast crisis (BC) compared with that in NBM CD34^+^ cells was analyzed. The data showed that *ZFX* expression was significantly higher in CML CD34^+^ cells than in NBM CD34^+^ cells, and *ZFX* expression was significantly higher in blast crisis patients than in chronic phase patients (Fig. 1A). Confocal analysis showed that ZFX protein had higher expression in CD34^+^ cells from a CML patient in CP than in normal control cells (Fig. 1B). However, the forced expression of BCR/ABL did not consistently increase ZFX expression in BaF3 cells or NBM CD34^+^ cells (Additional file 1: Fig. S1B–D).

To investigate whether ZFX enables malignant transformation or facilitates BCR/ABL induced leukemia, the murine nonmalignant BaF3 cells (depending on mIL-3) were utilized. As previous reports have shown that BCR/ABL transduction confers these cells growth factor independence in vitro [47], and the transduced cells are able to generate a leukemia mouse model readily [48]. Herein, CRISPR/dCas9 technology was employed to successfully increase Zfx expression in BaF3 cells (Fig. 1C). Zfx overexpression significantly promoted the growth and colony-forming cell (CFC) production of BaF3 cells in the presence of mIL-3 (Fig. 1D–E). However, Zfx overexpression did not support the growth of BaF3 cells in the absence of mIL-3 (data not shown). Next, overexpression of Zfx in BaF3–BCR/ABL cells was achieved by CRISPR/dCas9 technology and significantly increased the growth and CFC production of these cells (Fig. 1F–H). Imatinib mesylate (IM) treatment decreased the expression of ZFX in K562 cells and CML CD34^+^ cells in a dose-dependent manner (Additional file 1: Fig. S1E–F), suggesting that BCR/ABL maintained ZFX expression and ZFX played a role in the IM response of BCR/ABL^+^ cells. The control and Zfx-overexpressing BaF3-BCR/ABL cells were treated with IM, and the data showed that Zfx overexpression conferred IM resistance to BaF3-BCR/ABL cells (Fig. 1I). Finally, the control and Zfx-overexpressing BaF3 cells and BaF3–BCR/ABL cells were injected into lethally irradiated mice, and Kaplan–Meier analysis showed that Zfx overexpression significantly accelerated BCR/ABL-induced leukemia (Fig. 1J). However, Zfx-overexpressing group mice did not necessarily have increased BaF3–BCR/ABL cell infiltration than the control group mice (Additional file 1: Fig. S2), likely due to the relative small shortened latency caused by Zfx overexpression. Meanwhile, Zfx overexpression alone did not induce leukemia occurrence (Fig. 1J), which indicated that Zfx overexpression was not sufficient to cause malignant transformation of BaF3 cells in this study. Our data showed that increased expression of ZFX had the tendency to accelerate the generation of leukemia induced by BCR/ABL.

**Fig. 2:**
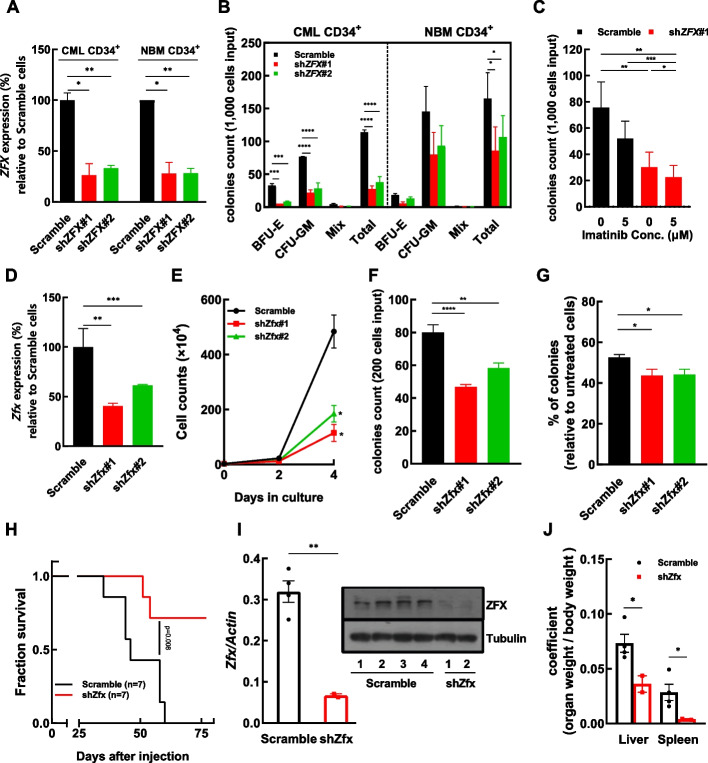
ZFX silencing inhibits CML CD34^+^ cells and sensitizes them to imatinib mesylate treatment. **A** Two independent shRNA sequences against *ZFX* were delivered into CML CD34^+^ cells (*n* = 3) or normal bone marrow (NBM) CD34^+^ cells (*n* = 3) with lentiviral vectors, and the relative expression of *ZFX* in shRNA-transduced cells compared with control (scramble) cells was measured by RT–qPCR. **B** The colony-forming cell (CFC) productions of ZFX-silenced and control cells were compared. BFU-E, burst-forming unit-erythroid; CFU-GM, colony-forming unit-granulocyte/macrophage; Mix, colony-forming unit-granulocyte, erythroid, macrophage, megakaryocyte. **C** ZFX-silenced and control CML CD34^+^ cells (*n* = 6) were plated for CFC assays with or without imatinib mesylate (IM). **D** Two independent shRNA sequences against *Zfx* were delivered into BaF3–BCR/ABL cells, and the relative expression of *Zfx* in shRNA-transduced cells compared with control cells was assessed by RT–qPCR. **E**, **F** The growth and CFC production of Zfx-silenced (shZfx) and control BaF–BCR/ABL cells were analyzed. **G** The CFC production of Zfx-silenced and control cells with or without IM was analyzed, and the percentage of CFC production relative to untreated cells was compared. **H** Zfx-silenced and control BaF–BCR/ABL cells were injected into lethally irradiated mice through the tail vein (seven mice in each group), and the survival of these mice was analyzed with the Kaplan–Meier method (log-rank test, **P < 0.01). **I** Leukemic cells were collected from the diseased mice of each group, and the expression of Zfx was analyzed by RT–qPCR and western blotting. **J** The weights of the liver and spleen from the diseased mice were measured, and the organ coefficient (organ weight/body weight, g/g) was calculated to evaluate the severity of leukemia symptom. Data are presented as the mean ± SEM, and Student’s *t*-test was used to estimate the *P*-values (*P < 0.05, **P < 0.01, and ***P < 0.001)

Conversely, the role of ZFX in CML cells was studied in a gene-silencing approach. Two independent shRNA sequences were delivered into CML CD34^+^ cells, and a similar experiment was conducted with NBM CD34^+^ cells as a control. The results showed that *ZFX* expression was inhibited effectively in both CML and NBM CD34^+^ cells to a similar extent (Fig. 2A), and ZFX silencing significantly suppressed the CFC production of CML CD34^+^ cells and NBM CD34^+^ cells. (Fig. 2B). It is worth noting that the suppression of CML cells was stronger than that of NBM cells induced by ZFX silencing (76% and 67% versus 47% and 35% inhibition). The differential effects of ZFX silencing on CML CD34^+^ cells versus NBM CD34^+^ cells were more pronounced, when a proliferation assay in liquid culture was conducted (Additional file 1: Fig. S3). The discrepancy between CFC and liquid culture assay was possibly due to the duration of these assays (14–16 days versus 4 days) and the different cytokine cocktails. In this study, several IM-resistant CML CD34^+^ cells (*n* = 6, CFC inhibition < 60% in the presence of 5 μM IM is considered as resistant samples following the criteria of Jiang’s study [49]) were collected to study the role of ZFX in the growth and IM response of these cells. The results showed that ZFX silencing significantly inhibited the CFC production of IM-resistant CD34^+^ cells. Although ZFX silencing did not sensitize these cells upon IM treatment, IM addition decreased the CFC production of ZFX-silenced CD34^+^ cells (Fig. 2C). Apoptosis of ZFX-silenced and control K562 cells upon IM treatment was assessed by flow cytometry, which indicated that ZFX silencing significantly enhanced cell apoptosis induced by IM (Additional file 1: Fig. S4). Moreover, Zfx silencing significantly decreased the growth and CFC production of BaF3–BCR/ABL cells (Fig. 2D–F) and significantly sensitized these cells upon IM treatment (Fig. 2G). Finally, Zfx-silenced and control BaF3–BCR/ABL cells were injected into lethally irradiated mice, and Kaplan–Meier analysis indicated that Zfx silencing significantly prolonged survival of mice (Fig. 2H). The diseased mice of each group were dissected to perform various analyses, when they were near death. RT–qPCR and western blotting showed that the leukemic cells from the Zfx-silenced group had lower Zfx expression than those from the control group (Fig. 2I). Zfx silencing significantly reduced coefficients of the liver and spleen (Fig. 2J). Flow cytometry analysis showed that Zfx silencing decreased BaF3-BCR/ABL cell infiltration in the peripheral blood (PB), bone marrow (BM), liver, and spleen (Additional file 1: Fig. S5), though the data did not reach statistical significance.

Overall, increased expression of ZFX in CML CD34^+^ cells modulates the growth and IM response of these cells, and ZFX promotes leukemogenesis induced by the BCR/ABL oncoprotein in a model of BaF3 cells.

### Microarray analysis reveals that ZFX regulates the expression of WNT3

**Fig. 3:**
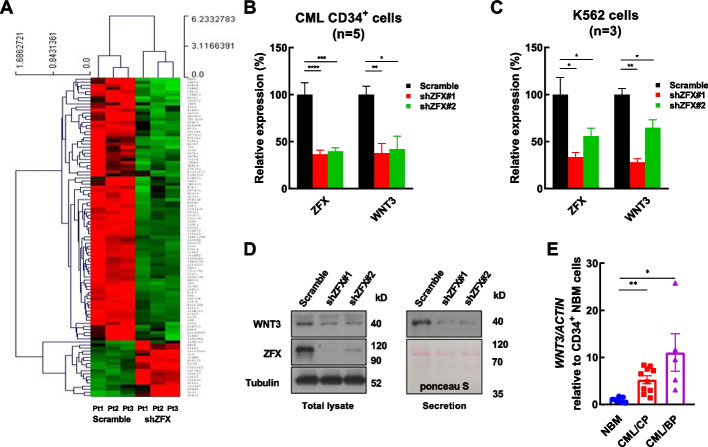
ZFX controls the expression of WNT3 in CML CD34^+^ cells. **A** The heatmap shows the differentially expressed transcripts (fold-change > 2 and *P* < 0.05) between ZFX-silenced CML CD34^+^ cells and control cells (*n* = 3). **B** The relative expression of *WNT3* was assessed by RT–qPCR in other ZFX-silenced CML CD34^+^ cells versus control cells (*n* = 5). **C** The expression of both *ZFX* and *WNT3* was measured in ZFX-silenced K562 cells versus control cells (*n* = 3). **D** Western blotting was conducted to analyze the expression of ZFX and WNT3 in ZFX-silenced and control K562 cells. The secreted protein from ZFX-silenced K562 cells and control cells was analyzed by western blotting. To monitor the sample loading, Ponceau S staining was performed. **E** The expression of *WNT3* was analyzed by RT–qPCR in CD34^+^ cells from normal bone marrow (NBM) of healthy donors (*n* = 8), CML patients in chronic phase (CP, *n* = 10), and those in blast crisis (BC, *n* = 5). Pt, patient. Data are presented as the mean ± SEM, and Student’s *t*-test was used to estimate the *P*-values (*P < 0.05 and **P < 0.01)

To delineate the molecular mechanism by which ZFX modulates the growth and IM response of CML stem/progenitor cells, ZFX-silenced and control CML CD34^+^ cells were analyzed by microarray (*n* = 3). In total, 103 differentially expressed transcripts were identified, with 18 upregulated transcripts and 85 downregulated transcripts upon ZFX silencing (Fig. 3A and Additional file 1: Table S6). Among these transcripts, *DIS3L* is a reported ZFX target transcript [8]; additionally, *SMO* and *HOXB5* are reported to regulate CML and normal stem/progenitor cells [50–52]. Therefore, these transcripts were selected for validation. The data showed that ZFX silencing significantly decreased the expression of *DIS3L*, *SMO*, and *HOXB5* in both CML CD34^+^ cells (*n* = 3) and K562 cells (Additional file 1: Fig. S6), which supported the validity of the microarray data in the present study.

Next, Kyoto Encyclopedia of Genes and Genomes (KEGG) enrichment analysis suggested that Wnt/β-catenin signaling was significantly perturbed upon ZFX silencing (Additional file 1: Fig. S7, Supplementary Table S7). RT–qPCR analysis showed that the expression of *WNT3* was significantly suppressed upon ZFX silencing in both CML CD34^+^ cells and K562 cells (Fig. 3B, C). In addition, the expression of WNT3 decreased upon ZFX silencing in the total protein extract and the secreted fraction of K562 cells (Fig. 3D). Finally, RT–qPCR analysis showed that *WNT3* had significantly higher expression in CD34^+^ cells from CML patients in both chronic phase and blast crisis than in NBM CD34^+^ cells (Fig. 3E). Interestingly, the expression pattern of *WNT3* was similar to that of *ZFX*. Taken together, these data indicated that ZFX activated WNT3 expression in CML cells.

### ZFX regulates WNT3 transcriptionally

**Fig. 4:**
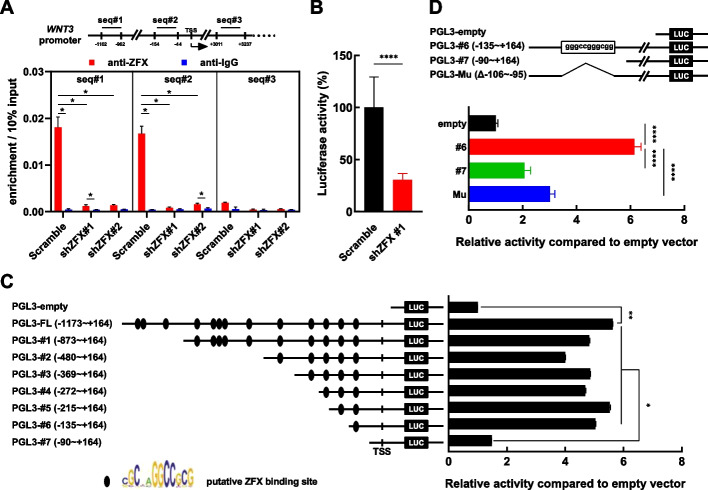
ZFX regulates the transcription of *WNT3*. **A** Chromatin immunoprecipitation (ChIP) was performed to analyze the interaction between ZFX protein and the *WNT3* gene. A schematic graph is displayed to show the primer sets designed for ChIP analysis (upper panel). The results of ChIP–qPCR analysis with various primer sets are shown (lower panel). **B** The promoter region of *WNT3* (−1137 to +164) was subcloned into a reporter vector (designated pGL3–FL), and this vector was transfected into ZFX-silenced and control (scramble) K562 cells. The relative luciferase activity of this reporter is shown. **C** pGL3–FL contained multiple putative ZFX binding sites; therefore, various deletion mutants were generated. Then, the activities of these vectors were assessed in K562 cells (*n* = 3). **D** One putative ZFX binding site (−106 to −95) was deleted to generate pGL3–Mu, and its activity was compared with that of pGL3#6 and pGL3#7 (*n* = 4), respectively. Data are presented as the mean ± SEM, and Student’s *t*-test was used to estimate the *P*-values (*P < 0.05, **P < 0.01, and ****P < 0.0001)

To determine whether ZFX regulates *WNT3* transcriptionally, chromatin immunoprecipitation (ChIP) was performed. Two sets of probes (seq#1 and seq#2) were designed in the promoter region of *WNT3,* and another set of probes (seq#3) was designed in the 3′ downstream of *WNT3* (Fig. 4A, upper panel). ChIP analysis showed that *WNT3* was detected by seq#1 or seq#2 but not seq#3 in K562 cells (Fig. S8). In addition, the control and ZFX silenced K562 cells were used to perform ChIP analysis in a quantitative manner, similar results were obtained (Fig. 4A, lower panel). Taken together, these data indicated that ZFX interacted with the promoter region of *WNT3*. Next, a piece of the promoter sequence of *WNT3* (full-length, FL) was subcloned to generate a reporter vector (pGL3–FL) for analyzing the promoter activity. The reporter activity of pGL3–FL in ZFX-silenced K562 cells was significantly lower than that in the control cells (Fig. 4B). To dissect the *WNT3* promoter, a series of reporter vectors were constructed (pGL3#1~#7), which contained various putative ZFX binding sites. The reporter activities of these vectors were measured and compared with that of the empty vector in K562 cells. The results showed that the FL promoter had transcriptional activity versus the empty control (~5.6-fold), and the deletion of the putative site (−106  to −95) nearest to the transcription starting site (TSS) caused a severe loss of transcriptional activity (Fig. 4C). A new reporter vector was then constructed to remove the putative site (−106 to −95) of pGL3#6, designated pGL3-Mu (Δ−106 to −95). Reporter analysis showed that both pGL3-Mu and pGL3#7 (without the −106 to −95 putative site) had significantly lower activity than pGL3#6 (only bearing the −106 to −95 putative site), which strongly indicated the importance of this site in the activation of *WNT3* by ZFX (Fig. 4D).

### A ZFX/WNT3 axis regulates the growth and IM response of BCR/ABL^+^ cells

**Fig. 5:**
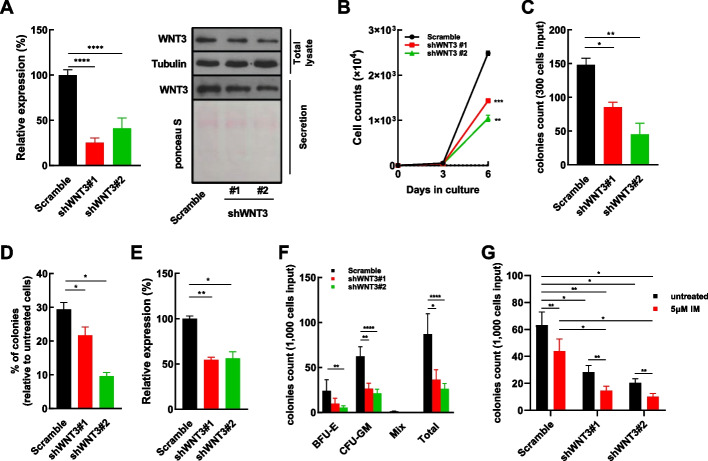
WNT3 silencing modulates the growth and imatinib mesylate response of CML cells. **A** Two independent shRNA sequences against *WNT3* were delivered into K562 cells with lentiviral vectors. The transcript expression of *WNT3* in shRNA-transduced and control cells were analyzed by RT–qPCR. The total lysate and secretion fraction of shRNA-transduced and control cells was analyzed by western blotting. Ponceau S staining was used to monitor the sample loading of the secreted proteins. **B**, **C** The growth and colony-forming cell (CFC) production of WNT3-silenced and control cells were analyzed (*n* = 3). **D** WNT3-silenced and control cells were plated for the CFC assay with or without imatinib mesylate (IM) (*n* = 3), and the percentage of surviving CFCs was calculated and compared (*n* = 3). **E** The expression of *WNT3* in shWNT3-transduced and control CML CD34^+^ cells was analyzed by RT–qPCR (*n* = 3). **F** CFC productions of WNT3-silenced and control CML CD34^+^ cells (*n* = 4) were compared. BFU-E, burst-forming unit-erythroid; CFU-GM, colony-forming unit-granulocyte/macrophage; Mix, colony-forming unit-granulocyte, erythroid, macrophage, megakaryocyte. **G** WNT3-silenced and control CML CD34^+^ cells (*n* = 5) were plated for CFC assays with or without IM. Data are presented as the mean ± SEM, and Student’s *t*-test was used to estimate the *P*-values (*P < 0.05, **P < 0.01, ***P < 0.001, and ****P < 0.0001)

As WNT3 expression was significantly higher in CML CD34^+^ cells than in NBM CD34^+^ cells (Fig. 3E), its role in CML cells was studied. Two independent shRNA sequences were validated to silence both the transcript and protein expression of WNT3 in K562 cells. In addition, the secreted WNT3 was reduced upon gene silencing (Fig. 5A). WNT3 silencing significantly inhibited the growth and CFC production of K562 cells (Fig. 5B, C) and enhanced the IM response of these cells (Fig. 5D). Additionally, WNT3 silencing inhibited CFC production of CML CD34^+^ cells (Fig. 5E, F) and sensitized these cell to IM treatment (Fig. 5G).

**Fig. 6:**
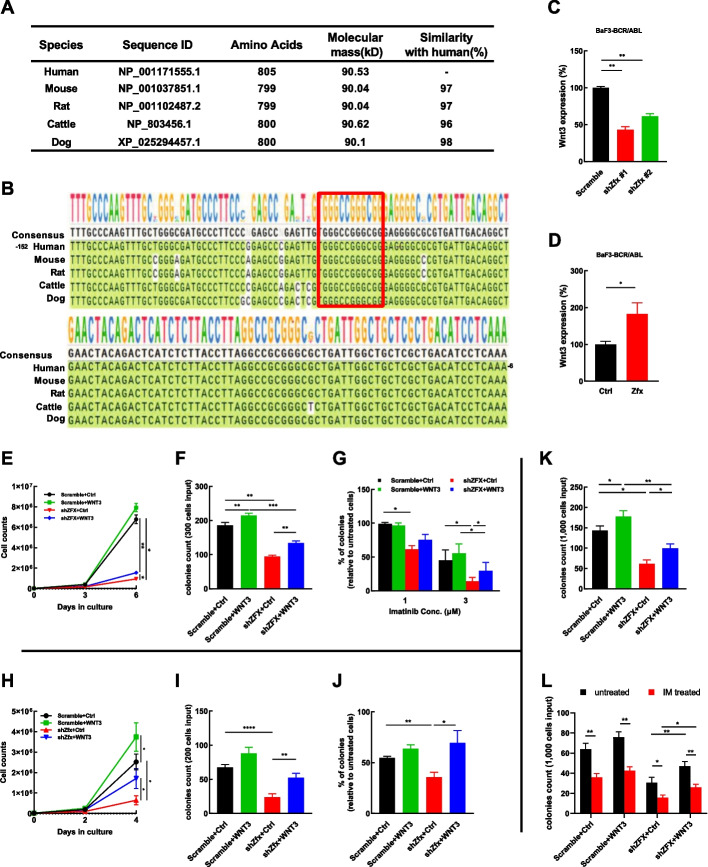
A conserved ZFX/WNT3 axis modulates the growth and imatinib response mesylate of BCR/ABL^+^ cells. **A** The similarity analysis of ZFX proteins of various species is shown. **B** The promoter regions of WNT3 of various species were subjected to alignment analysis. A key ZFX binding site (GGGCCGGGCGG) is highlighted in the red box. **C** The relative expression of *Wnt3* in BaF3–BCR/ABL cells upon Zfx silencing was analyzed by RT–qPCR. **D** The relative expression of *Wnt3* in BaF3–BCR/ABL cells upon Zfx overexpression was assessed by RT–qPCR. **E**–**G** WNT3 and the empty control (Ctrl) were delivered into the control (Scramble) and ZFX-silenced K562 cells, the growth (*n* = 4), CFC production (*n* = 4), and imatinib mesylate (IM) response (*n* = 3) of variously transduced cells were measured. **H**–**J** WNT3 and the empty control (Ctrl) were delivered into the control (scramble) and Zfx-silenced BaF3–BCR/ABL cells, the growth, CFC production, and IM response of variously transduced cells were measured (*n* = 4). **K**, **L** WNT3 and the empty control (Ctrl) were delivered into the control (scramble) and ZFX-silenced CML CD34^+^ cells, CFC production and IM response of variously transduced cells were measured (*n* = 4). Data are presented as the mean ± SEM, and Student’s *t*-test was used to estimate the *P*-values (*P < 0.05, **P < 0.01, ***P < 0.001, and ****P < 0.0001)

Next, we asked whether the ZFX/WNT3 regulatory axis is conserved. First, alignment analysis was performed with Needleman–Wunsch algorithm, which showed that human ZFX protein had high similarity to several other mammalian ZFX proteins, including murine Zfx protein (Fig. 6A, Additional file 1: Fig. S9A). Next, the sequences of the WNT3 promoter region from several mammals were compared. The results showed high similarity among these regulatory sequences, and the ZFX binding site (GGGCCGGGCGG) was conserved among all sequences we queried (Fig. 6B), which suggested that the ZFX/WNT3 regulatory axis is conserved. Then, the expression of *Wnt3* in BaF3–BCR/ABL cells upon Zfx manipulation was studied. The results showed that Zfx silencing inhibited the expression of *Wnt3* (Fig. 6C), whereas Zfx overexpression enhanced the expression of *Wnt3* (Fig. 6D).

Then, a rescue experiment was performed to study the role of WNT3 in ZFX silencing-induced growth inhibition and IM hypersensitivity. WNT3 was overexpressed in both ZFX-silenced and control K562 cells, and the results showed that WNT3 overexpression did not necessarily alter the growth or IM response of the control cells, whereas this action partially rescued the effects of ZFX silencing on the growth, CFC production, and IM response of these cells (Fig. 6E–G, Additional file 1: Fig. S10A). We continued to ask whether WNT3 plays a role in Zfx silencing–modulated growth of BaF3–BCR/ABL cells. Alignment analysis showed that human WNT3 protein had high similarity (99%) to murine Wnt3 protein (Additional file 1: Fig. S9B), which allowed us to overexpress human WNT3 in BaF3 cells to address the above question. Western blotting showed that WNT3 was successfully overexpressed in Zfx-silenced and control BaF3–BCR/ABL cells (Additional file 1: Fig. S10B), and this action partially rescued the inhibitory effects of Zfx silencing on the growth, CFC production, and the IM response of these cells (Fig. 6H–J). Importantly, WNT3-overexpression also rescued the CFC production and IM response of CML CD34^+^ cells (n = 4) (Fig. 6K, L).Thus, the current study demonstrated that a conserved ZFX/WNT3 axis modulates the growth and IM response of BCR/ABL^+^ cells.

### ZFX silencing attenuates Wnt/β-catenin signaling in BCR/ABL^+^ cells

**Fig. 7:**
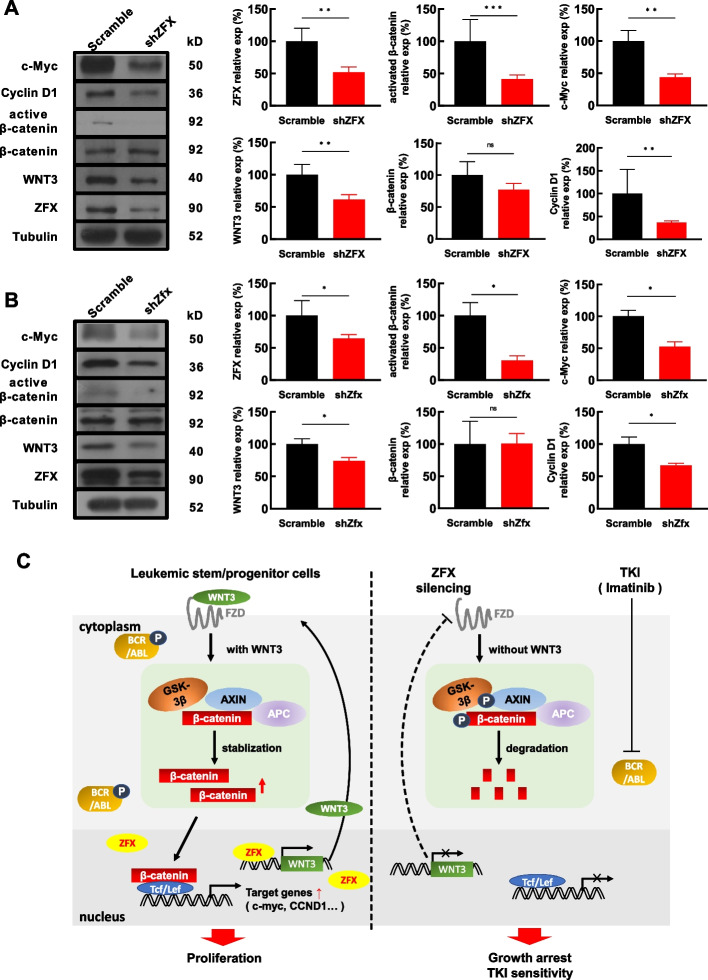
ZFX silencing attenuates β-catenin signaling in BCR/ABL^+^ cells. **A** In K562 cells, the effects of ZFX silencing on several molecules in the Wnt signaling pathway were analyzed by western blotting, and representative graphs are shown (left). Cumulative data from the blots were analyzed statistically (right, *n* ≥ 3). **B** In BaF3–BCR/ABL cells, the expression of several molecules in the Wnt signaling pathway upon Zfx silencing was analyzed by western blotting (left), and cumulative data from the blots were subjected to statistical analysis (right, *n* ≥ 3). Data are presented as the mean ± SEM, and Student’s *t*-test was used to estimate the *P*-values (*P < 0.05 and **P < 0.01). **C** The proposed model illustrates the importance of the ZFX/WNT3 axis in CML stem/progenitor cells. The elevated ZFX promotes the transcription of *WNT3*, which sustains β-catenin signaling in CML stem/progenitor cells and confers growth advantage and drug resistance to these cells. FZD, Frizzled receptors

To investigate the effects of ZFX silencing on Wnt/β-catenin signaling, the expression of a few molecules was analyzed by western blotting in K562 cells. ZFX silencing did not affect the expression of β-catenin; however, the expression of activated β-catenin was significantly decreased (Fig. 7A). The decreased expression of activated β-catenin was also confirmed by flow cytometry (Additional file 1: Fig. S11A). Consequently, the expression of c-MYC and cyclin D1, the two typical downstream targets of β-catenin signaling, were also significantly decreased (Fig. 7A). Similar results were obtained with BaF3–BCR/ABL cells when comparing Zfx-silenced cells with control cells (Fig. 7B, Additional file 1: Fig. S11B). In addition, the transcript expression of *c-MYC* and *CCND1* was measured in both K562 and CML CD34^+^ cells upon ZFX silencing. The data showed that *CCND1* expression was consistently decreased upon ZFX silencing (Additional file 1: Fig. S12A, B). The regulatory effects of Zfx on the transcript expression of *c-Myc* and *Ccnd1* in both BaF3 cells and BaF3–BCR/ABL cells were confirmed with RT–qPCR (Additional file 1: Fig. S12C-S12D). The decreased expression of cyclin D1 was in line with our previous report that ZFX silencing causes significant G0/G1 arrest in K562 cells [18]. Taken together, the present study revealed a pivotal role of the ZFX/WNT3 axis in CML stem/progenitor cells, which activates WNT3/β-catenin signaling to promote the growth of these cells and confer IM resistance to these cells (Fig. 7C).

## Discussion

The upregulation of ZFX in CML cells has been reported [17, 18]; however, whether it is deregulated in CML stem/progenitor cells has not yet been reported. In the present study, we showed that *ZFX* was highly expressed in CD34^+^ cells from CML patients in both chronic phase and blast crisis; moreover, *ZFX* expression was higher in blast crisis patients than in chronic phase patients. Gene silencing experiments demonstrated that ZFX played a critical role in the growth and IM response of these cells. Therefore, our data provided another example in which ZFX modulates the properties of cancer stem cells, in agreement with other reports [10, 21, 22]. ZFX is upregulated in numerous human cancers; however, whether ZFX alone is sufficient to cause malignant transformation is not clear yet. In the present study, our data showed that ZFX promoted the growth of BaF3 cells (immortalized but nonmalignant cells) in vitro with mIL-3; however, ZFX alone did not confer BaF3 cells cytokine independence or enable leukemia generation in mice. Considering that ZFX silencing inhibited the growth of CML cells, our data indicated that ZFX facilitates leukemogenesis induced by BCR/ABL similar to its role in other tumor models induced by different oncoproteins, such as Hedgehog or MLL–AF9 [8, 16].

It has been reported that ZFX plays an important role in CML cells by altering signaling pathways, such as the PI3K/AKT pathway and B4GALT1-mediated glycosylation [17, 18]. However, the direct target of ZFX has not yet been identified in CML cells. Herein, we generated a gene expression profile comparing ZFX-silenced CML CD34^+^ cells with their controls. Among the differentially expressed transcripts, the Wnt/β-catenin pathway was enriched in KEGG analysis. It is well known that the Wnt/β-catenin pathway plays a critical role in hematological malignancies, including CML [28]. Moreover, ZFX silencing reduces the transactivation of β-catenin in liver EpCAM^+^ cancer stem cells; however, the detailed mechanism remains unclear [22]. Our data showed that WNT3 was upregulated in CD34^+^ cells from CML patients in both CP and BC, which agreed with a previous report that WNT3 was highly expressed in CML patients in BC [53]. We found that WNT3 sustained the growth of these cells and conferred IM resistance to these cells. To the best of our knowledge, this is the first report about the increased expression and functional role of WNT ligand in CML stem/progenitor cells. Previous reports have shown that autocrine cytokines, such as IL-3, G-CSF, TNFα, and GM-CSF, play an important role in CML pathology and TKI response [54–56]. Herein, our study provides another example of autocrine signaling in CML stem/progenitor cells. In line with previous reports [32–43], our data also support the crucial role of WNT/β-catenin in CML stem/progenitor cells and promote the notion that the combination of WNT inhibitor (e.g., WNT974) and TKIs provides a new option for disease treatment [43]. Unfortunately, there is no specific inhibitor against WNT3 yet, which precludes a potential assessment of the efficacy of the combination of WNT3 inhibitor and TKIs. A rescue experiment demonstrated that ZFX silencing modulated CML cells partially through WNT3. We also found that ZFX silencing decreased the expression of c-Myc and cyclin D1, suggesting decreased WNT signaling upon ZFX silencing.

## Conclusions

Our data identified a conserved ZFX/WNT3 axis, which controls the growth and IM response of CML stem/progenitor cells through a canonical β-catenin signaling. The present study deepens the understanding of CML pathology and possibly provides new clues to improve disease management.

### Supplementary Information

**Additional file 1: Table S1. **The clinical characteristics of chronic myeloid leukemia patients recruited in this study. **Table S2.** The shRNA sequences used in this study. **Table S3.** The gene-specific primers used in this study. **Table S4.** Antibodies used in this study. **Table S5.** Guide RNA used in this study. **Table S6.** Differentially expressed transcripts between ZFX-silenced CML CD34^+ ^cells and their controls. **Table S7.** KEGG analysis of signaling pathways among differentially expression transcripts comparing ZFX-silenced CML CD34^+ ^cells with their controls. **Figure S1.** Zinc finger protein X-linked is upregulated in chronic myeloid leukemia cells and decreases upon imatinib methylate treatment. **Figure S2.** Zfx overexpression promotes leukemia generation induced by BaF3-BCR/ABL cells. **Figure S3.** ZFX silencing specifically inhibits chronic myeloid leukemia CD34^+^ cells but not normal bone marrow CD34^+^ cells in liquid culture. **Figure S4.** ZFX silencing promtes Imatinib mesylate induced cell death of K562 cells. **Figure S5.** Zfx silencing decreases the infiltration of BaF3-BCR/ABL cells. **Figure S6.** The expression of *DIS3L*, *SMO*, and *HOXB5* in ZFX-silenced and control CML cells. **Figure S7.** The KEGG analysis of the differentially expressed transcripts comparing ZFX-silenced with control CML CD34^+^ cells. **Figure S8.** Chromatin immunoprecipitation (ChIP) was performed to analyze the interaction between ZFX protein and the *WNT3* gene. **Figure S9.** The alignment analysis of ZFX and WNT3 proteins in mammals. **Figure S10.** The expression of WNT3 and ZFX in variously transduced K562 and BaF3-BCR/ABL cells. **Figure S11.** Activated β-catenin was significant reduced upon ZFX silencing in both K562 and BaF3-BCR/ABL cells. **Figure S12.** The expression of *c-MYC* and *CCND1* is regulated by ZFX in various cellular models.

## Data Availability

Data supporting the findings are included in this article and the Additional file. Microarray data are deposited in GEO and assigned an accession number as GSE241116. Materials are available from the corresponding authors on reasonable request.

## Abbreviations

BC: Blast crisis
CFC: Colony-forming cell
ChIP: Chromatin immunoprecipitation
CML: Chronic myeloid leukemia
CP: Chronic phase
EPO: Erythropoietin
FBS: Fetal bovine serum
FZD: Frizzled
G-CSF: Granulocyte colony-stimulating factor
GM-CSF: Granulocyte macrophage colony-stimulating factor
IL-3: Interleukin-3
IL-6: Interleukin-6
IM: Imatinib mesylate
NBM: Normal bone marrow
RT–qPCR: Real-time quantitative PCR
SCF: Stem cell factor
TKI: Tyrosine kinase inhibitor
ZFX: Zinc finger protein X-linked
