# Supplementary material for: A conserved ZFX/WNT3 axis modulates the growth and imatinib response of chronic myeloid leukemia stem/progenitor cells
Source: Cell Mol Biol Lett. 2023 Oct 20;28:83. doi: 10.1186/s11658-023-00496-z (PMC10589942; doi:10.1186/s11658-023-00496-z)
Supplement: Supplementary file 1 — Additional file 1: Table S1. The clinical characteristics of chronic myeloid leukemia patients recruited in this study. Table S2. The shRNA sequences used in this study. Table S3. The gene-specific primers used in this study. Table S4. Antibodies used in this study. Table S5. Guide RNA used in this study. Table S6. Differentially expressed transcripts between ZFX-silenced CML CD34+ cells and their controls. Table S7. KEGG analysis of signaling pathways among differentially expression transcripts comparing ZFX-silenced CML CD34+ cells with their controls. Figure S1. Zinc finger protein X-linked is upregulated in chronic myeloid leukemia cells and decreases upon imatinib methylate treatment. Figure S2. Zfx overexpression promotes leukemia generation induced by BaF3-BCR/ABL cells. Figure S3. ZFX silencing specifically inhibits chronic myeloid leukemia CD34+ cells but not normal bone marrow CD34+ cells in liquid culture. Figure S4. ZFX silencing promtes Imatinib mesylate induced cell death of K562 cells. Figure S5. Zfx silencing decreases the infiltration of BaF3-BCR/ABL cells. Figure S6. The expression of DIS3L, SMO, and HOXB5 in ZFX-silenced and control CML cells. Figure S7. The KEGG analysis of the differentially expressed transcripts comparing ZFX-silenced with control CML CD34+ cells. Figure S8. Chromatin immunoprecipitation (ChIP) was performed to analyze the interaction between ZFX protein and the WNT3 gene. Figure S9. The alignment analysis of ZFX and WNT3 proteins in mammals. Figure S10. The expression of WNT3 and ZFX in variously transduced K562 and BaF3-BCR/ABL cells. Figure S11. Activated β-catenin was significant reduced upon ZFX silencing in both K562 and BaF3-BCR/ABL cells. Figure S12. The expression of c-MYC and CCND1 is regulated by ZFX in various cellular models. [file 11658_2023_496_MOESM1_ESM.pdf]

Additional file

A conserved ZFX/WNT3 axis modulates the growth and imatinib response of chronic myeloid leukemia stem/progenitor cells

Xiuyan Zhang[1,2,†], Yu Wang[1], Jinchang Lu[1], Lun Xiao[3], Hui Chen[1], Quanxue Li[4], Yuan-Yuan Li[4], Peng Xu[1], Changgeng Ruan[2,5,6,7], Haixia Zhou[2,5,7,†], Yun Zhao[1,5,6,7,†]

[1] Cyrus Tang Medical Institute, Soochow University, Suzhou 215123, China, [2] The First Affiliated Hospital of Soochow University, Jiangsu Institute of Hematology, NHC Key Laboratory of Thrombosis and Hemostasis, Suzhou 215006, China, [3] Department of Vascular Surgery, The Affiliated Drum Tower Hospital, Nanjing University Medical School, Nanjing 210008, China, [4] Shanghai-MOST Key Laboratory of Health and Disease Genomics, Shanghai Institute for Biomedical and Pharmaceutical Technologies, Shanghai 200237, China, [5] National Clinical Research Center for Hematologic Diseases, Suzhou 215006, China, [6] Collaborative Innovation Center of Hematology, Soochow University, Suzhou 215006, China, [7] MOE Engineering Center of Hematological Disease, Soochow University, Suzhou 21513, China.

[†], these authors are co-corresponding authors.

Table S1

The clinical characteristics of chronic myeloid leukemia patients recruited in this study.

Patient No.	Age	Gender	WBC (×10^9/L)	Hb (g/L)	Plt (×10^9/L)	Disease stage
1	48	M	220.9	102	373	CP
2	42	M	27.6	152	210	CP
3	54	F	201	108	228	CP
4	28	M	494.16	78	327	CP
5	47	M	48.54	141	480	CP
6	23	M	189.4	108	355	CP, IM R
7	75	M	69.7	135	685	CP
8	36	M	86.93	110	508	CP
9	33	F	73.55	109	165	BC
10	40	M	25.62	84	223	BC
11	58	M	26.48	99	49	BC
12	38	M	34.95	115	90	BC
13	42	M	15.05	62	49	BC
14	52	M	17.37	85	347	BC
15	23	F	126.81	67	19	BC
16	38	M	20	91	50	BC
17	9	M	206	102	259	CP
18	50	F	193	100	180	CP
19	73	F	82.5	107	144	CP
20	53	M	37.78	144	411	CP
21	51	M	175.83	103	233	CP
22	32	M	180.89	125	272	CP
23	41	M	137.11	109	349	CP
24	28	M	89.76	121	120	CP
25	N/A	N/A	N/A	N/A	N/A	CP, IM R
26	43	M	299	83	1218	CP, IM R
27	43	M	195.1	131	181	CP, IM R
28	61	F	66.04	109	263	CP, IM R
29	42	M	110	100	394	CP, IM R
30	29	F	303	98	698	CP
31	58	F	N/A	N/A	N/A	CP
32	48	F	56.7	134	359	CP
33	37	F	242.65	101	944	CP
34	63	F	245.58	77	234	CP
35	N/A	N/A	N/A	N/A	N/A	CP
36	43	F	97.3	120	427	CP

37	37	M	147.53	104	466	CP
38	53	M	62.1	146	319	CP
39	27	M	196.57	124	898	CP
40	31	M	765	N/A	145	CP
41	28	M	62.1	146	319	CP
42	21	F	182.13	91	399	CP
43	18	F	286.75	85	769	CP
44	34	M	40.5	112	131	CP
45	31	F	35.59	142	142	CP
46	75	M	28.23	134	605	CP
47	65	M	26.48	138	224	CP
48	47	F	242	95	407	CP
49	71	M	31	N/A	N/A	CP
50	84	M	45.81	108	207	CP
51	54	F	159.84	101	331	CP
52	72	M	45.4	107	161	CP
53	71	M	198	N/A	N/A	CP
54	73	F	81.83	147	16	CP
55	37	M	236.3	100	353	CP
56	54	M	160.2	115	335	CP
57	79	M	9.85	141	188	CP

CP: Chronic phase; BC: Blast crisis; IM R: Imatinib resistance; N/A: not available.

Table S2

The shRNA sequences used in this study.

**name**	**sequence（5'-3'）**
Scramble	TTCTCCGAACGTGTCACGT
shZFX #1	GTCGGAAATTGATCCTTGTAA
shZFX #2	CCAATCAGTCTCATTCACATA
shWNT3 #1	CCAGGAGTGTATTCGCATCTAC
shWNT3 #2	CGGCTGTGACTCGCATCATAA
shZfx #1	GCCTATTGAATCGCCATCTTT
shZfx #2	ACAGAAATTGACCCTTGTAAA

Table S3

The gene-specific primers used in this study.

Name		sequence（5’-3’）
**For RT-qPCR**		
Human *ACTIN*	F	CACCATTGGCAATGAGCGGTTCC
	R	GTAGTTTCGTGGATGCCACAGG
Human/Mouse *ZFX*	F	AACCTTCATGCCGATTGCATG
	R	CCGGTTTTCAATTCCATCAGAAT
Human *WNT3*	F	GCGTGTTAGTGTCCAGGGAGTT
	R	TGAGGTGCATGTGGTCCAGGAT
Human *c-MYC*	F	CTGGTGCTCCATGAGGAGAC
	R	AGACTCTGACCTTTTGCCAGG
Human *CCND1*	F	TCTACACCGACAACTCCATCCG
	R	TCTGGCATTTTGGAGAGGAAGTG
Mouse *Actin*	F	GAG ACC TTC AAC ACC CCA GC
	R	ATGTCACGCACGATTTCCC
Mouse *Wnt3*	F	GCAGTGCATGAACAGCAAGTG
	R	GCCATTCCGGTTTTCAATTCC
Mouse *c-Myc*	F	TCGCTGCTGTCCTCCGAGTCC
	R	GGTTTGCCTCTTCTCCACAGAC
Mouse *Ccnd1*	F	GGGATGTGAGGGAAGAGGTGA
	R	GCAGCGAAAACAACGTGAAA
**For ChIP assay**		
Seq #1	F	ACCTGACCATGATGTCAATACC
	R	ACCAAATGCCTCCAGTTACC
Seq #2	F	GCTTTGCCCAAGTTTGCT
	R	GCCTAAGGTAAGAGATGAGTCTG
Seq #3	F	GGGCACAGGCTTCCTTGACACCAGC
	R	GTACTGGAATCTGACTGCCAGGCGGG
**For WNT3 promoter clone**		
pGL3-FL (-1137~+164)	F	*ACGCGT*AAAGGCACCCAGGTGACCGG
pGL3-#1 (-873~+164)	F	*ACGCGT*AGTGAGGGGCGCTCCGGA
pGL3-#2 (-480~+164)	F	*ACGCGT*CGAGAGGCCAGAGAGCGG
pGL3-#3 (-369~+164)	F	*ACGCGT*CTTTCTACCCTCGATTCCTC
pGL3-#4 (-272~+164)	F	*ACGCGT*ATGTGAACGCCCGGGTCAAA

pGL3-#5 (-215~+164)	F	*ACGCGT*GCGGTAGCAGAGGGTCCG
pGL3-#6 (-135~+164)	F	*ACGCGT*GGCGATGCCCTTCCCGG
pGL3-#7 (-90~+164)	F	*ACGCGT*GCGCGTGATTGACAGGCTGAA
pGL3-WNT3	R	*AGATCT*ATTAGAAGAGGCGCCGAGGA
pGL3-Mu (△-106~-95)	F	TTCCCGGAGCCCGAGTTGTAGGGGCGCGTGATTGACA
	R	TGTCAATCACGCGCCCCTACAACTCGGGCTCCGGGAA
**For WNT3 overexpression**		
Human *WNT3*	F	*TCTAGA*ATGGAGCCCCACCTGCTCGGGCT
	R	*CATATG*CTTGCAGGTGTGCACGTCGTAGATG

The underlined sequences represent restriction endonuclease sites.

Table S4

Antibodies used in this study.

Antibody	*Vendor*	*Catalog Number*
ZFX	Origene	TA319738
WNT3	Abcam	ab116222
c-Myc	Abcam	ab32072
cyclin D1	ABclonal	A19038
β-catenin	Cell Signaling Technology	9562
activated β-catenin	Cell Signaling Technology	8814
Tubulin	Multisciences	ab009

Table S5

Guide RNA used in this study.

**name**		**sequence（5’-3’）**
Control	F	*CACC*GAAGATGGGCGGGAGTCTTC
	R	*AAAC*GAAGACTCCCGCCCATCTTC
Zfx	F	*CACC*GACCCTCGCGGAGAGGCGGT
	R	*AAAC*ACCGCCTCTCCGCGAGGGTC

Table S6

Differentially expressed transcripts between ZFX-silenced CML CD34$^+$ cells and their controls.

Accession	GeneSymbol	FC	*P* value	Gene description
**Down-regulated upon ZFX silencing**				
NM_001304441	*MMP8*	0.139909	1.08E-05	matrix metallopeptidase 8, transcript variant 2
NM_019055	*ROBO4*	0.19619	7.5E-05	roundabout guidance receptor 4, transcript variant 1
NM_024812	*BAALC*	0.212218	0.000428	brain and acute leukemia, cytoplasmic, transcript variant 1
NM_001882	*CRHBP*	0.23116	0.001566	corticotropin releasing hormone binding protein
NM_001077657	*SMCO1*	0.239011	0.003687	single-pass membrane protein with coiled-coil domains 1
NM_015656	*KIF26A*	0.245145	0.000175	kinesin family member 26A
NM_182511	*CBLN2*	0.267238	0.001162	cerebellin 2 precursor
NM_001282658	*CCDC3*	0.290599	6.02E-06	coiled-coil domain containing 3, transcript variant 2
NM_152751	*BEND7*	0.29112	2.05E-05	BEN domain containing 7, transcript variant 1
NM_138455	*CTHRC1*	0.307097	0.00016	collagen triple helix repeat containing 1, transcript variant 1
NM_014548	*TMOD2*	0.319775	0.002105	tropomodulin 2, transcript variant 1
NM_080743	*SRSF12*	0.33305	2.49E-05	serine/arginine-rich splicing factor 12

NM_002487	*NDN*	0.339617	4.46E-05	necdin, melanoma antigen (MAGE) family member
NM_002514	*NOV*	0.347324	0.004467	nephroblastoma overexpressed
NM_012092	*ICOS*	0.351486	2.68E-05	inducible T-cell co-stimulator
NM_033446	*MVB12B*	0.366645	0.001122	multivesicular body subunit 12B, transcript variant 1
NM_001282402	*MIXL1*	0.367065	0.000299	mix paired-like homeobox, transcript variant 1
NM_001005386	*ACTR2*	0.372055	3.48E-07	ARP2 actin-related protein 2, transcript variant 1
NM_002163	*IRF8*	0.379491	0.01554	interferon regulatory factor 8
NM_001257097	*RHNO1*	0.393294	1.68E-07	RAD9-HUS1-RAD1 interacting nuclear orphan 1, transcript variant 4
NM_001199707	*KIAA0895*	0.405318	0.007738	KIAA0895, transcript variant 4
NM_021267	*CERS1*	0.406715	3.79E-05	ceramide synthase 1, transcript variant 1
NM_020932	*MAGEE1*	0.406791	0.001093	melanoma antigen family E1
NM_020311	*ACKR3*	0.409682	0.013435	atypical chemokine receptor 3
NM_001134453	*DSG4*	0.414142	0.010746	desmoglein 4, transcript variant 1
NM_198525	*KIF7*	0.421939	0.000209	kinesin family member 7
NM_001291324	*BAHCC1*	0.424231	0.004491	BAH domain and coiled-coil containing 1
NM_001408	*CELSR2*	0.424612	0.002815	cadherin EGF LAG seven-pass G-type receptor 2
NM_015566	*FAM169A*	0.424789	9.71E-05	family with sequence similarity 169 member A, transcript variant 1

NM_033046	*RTKN*	0.424932	0.038573	Rhotekin, transcript variant 2
NM_145263	*SPATA18*	0.425332	0.000215	spermatogenesis associated 18, transcript variant 1
NM_024657	*MORC4*	0.427847	0.002273	MORC family CW-type zinc finger 4, transcript variant 1
NM_003918	*GYG2*	0.432299	9.69E-05	glycogenin 2, transcript variant 2
NM_018092	*NETO2*	0.432299	0.013472	neuropilin (NRP) and tolloid (TLL) -like 2, transcript variant 1
NM_001145206	*KIAA1671*	0.43446	0.01119	KIAA1671
NM_145804	*ABTB2*	0.436317	0.00066	ankyrin repeat and BTB (POZ) domain containing 2
NM_000892	*KLKB1*	0.438068	0.011494	kallikrein B, plasma (Fletcher factor) 1
NM_001145418	*TTC28*	0.440821	0.002232	tetratricopeptide repeat domain 28
NM_002333	*LRP3*	0.441352	0.009321	low density lipoprotein receptor-related protein 3
NM_003015	*SFRP5*	0.44161	0.026326	secreted frizzled-related protein 5
NM_001243428	*ERG*	0.44286	0.000213	ETS (erythroblast transformation-specific) transcription factor ERG, transcript variant 5
NM_001286389	*TMEM179*	0.444102	0.000199	transmembrane protein 179, transcript variant 1
NM_032211	*LOXL4*	0.447152	9.62E-05	lysyl oxidase-like 4
NM_001844	*COL2A1*	0.447891	0.00465	collagen type II alpha 1 chain, transcript variant 1
NM_174899	*FBXO36*	0.449311	0.000623	F-box protein 36

NM_001159704	*FHL1*	0.451949	0.001046	four and a half LIM domains 1, transcript variant 4
NM_001201377	*ALDH7A1*	0.453034	0.000199	aldehyde dehydrogenase 7 family member A1, transcript variant 1
NM_006937	*SUMO2*	0.453618	2.39E-05	small ubiquitin-like modifier 2, transcript variant 1
NM_005506	*SCARB2*	0.457932	0.000305	scavenger receptor class B member 2, transcript variant 1
NM_023073	*C5orf42*	0.45932	0.000587	chromosome 5 open reading frame 42, ciliogenesis and planar polarity effector 1
NM_032801	*JAM3*	0.461534	6.97E-09	junctional adhesion molecule 3, transcript variant 1
NM_022783	*DEPTOR*	0.462386	0.000106	DEP domain containing MTOR-interacting protein, transcript variant 1
NM_033315	*RASL10B*	0.462962	0.001356	RAS-like family 10 member B
NM_019020	*TBC1D16*	0.463293	0.000684	TBC1 domain family member 16, transcript variant 1
NM_024792	*FAM57A*	0.464997	1.43E-05	family with sequence similarity 57 member A
NM_001018072	*BTBD11*	0.465224	0.037851	BTB (POZ) domain containing 11, transcript variant a
NM_001143830	*GAS2*	0.465316	0.011994	growth arrest-specific 2, transcript variant 3
NM_006822	*RAB40B*	0.467079	0.020368	RAB40B, member RAS oncogene family
NM_004155	*SERPINB9*	0.46776	0.000768	serpin family B member 9
NM_002147	*HOXB5*	0.468836	0.006081	homeobox B5
NM_000852	*GSTP1*	0.46983	0.001126	glutathione S-transferase pi 1

NM_001143688	*DIS3L*	0.471546	5.37E-05	DIS3 like exosome 3'-5' exoribonuclease, transcript variant 1
NM_015404	*DFNB31*	0.474001	0.036919	WHRN, hirlin, transcript variant 1
NM_017843	*BCAS4*	0.474171	0.01101	breast carcinoma amplified sequence 4, transcript variant 1
NM_001185095	*AIF1L*	0.474925	0.001362	allograft inflammatory factor 1-like, transcript variant 3
NM_006849	*PDIA2*	0.475899	0.001335	protein disulfide isomerase family A member 2
NM_032148	*SLC41A2*	0.476591	0.001382	solute carrier family 41 member 2
NM_001010911	*CASC10*	0.476705	0.000621	MIR1915 host gene
NM_198567	*SIMC1*	0.477432	6E-07	SUMO-interacting motifs containing 1
NM_001130675	*CLGN*	0.478103	0.000835	calmegin, transcript variant 2
NM_015189	*EXOC6B*	0.478958	2.08E-05	exocyst complex component 6B
NM_001110514	*EBF4*	0.480615	0.020092	early B-cell factor (EBF) family member 4
NM_030753	*WNT3*	0.481848	0.01957	Wnt family member 3
NM_001197294	*DPYSL3*	0.48318	0.007079	dihydropyrimidinase-like 3, transcript variant 1
NM_016269	*LEF1*	0.484753	0.003438	lymphoid enhancer-binding factor 1, transcript variant 1
NM_177454	*FAM171B*	0.486888	0.002115	family with sequence similarity 171 member B
NM_138983	*OLIG1*	0.487464	0.012193	oligodendrocyte transcription factor 1
NM_024768	*EFCC1*	0.488661	0.001055	EF-hand and coiled-coil domain containing 1

NM_016331	*ZNF639*	0.489349	1.97E-05	zinc finger protein 639, transcript variant 1
NM_032829	*FAM222A*	0.490863	0.034554	family with sequence similarity 222 member A
NM_032373	*PCGF5*	0.491016	0.000209	polycomb group ring finger 5, transcript variant 1
NM_001178083	*EXT2*	0.492444	6.4E-05	exostosin glycosyltransferase 2, transcript variant 3
NM_005631	*SMO*	0.497555	1.96E-05	Smoothened, frizzled class receptor
NM_001004759	*OR51T1*	0.498773	0.007565	olfactory receptor family 51 subfamily T member 1
NM_016613	*FAM198B*	0.499148	0.048816	family with sequence similarity 198 member B, transcript variant 2
**Up-regulated upon ZFX silencing**				
NM_173637	*SLC25A41*	2.013162	0.011278	solute carrier family 25 member 41
NM_001074	*UGT2B7*	2.040067	0.028442	UDP glucuronosyltransferase family 2 member B7
NM_001128850	*RRAD*	2.074645	0.017989	Ras related glycolysis inhibitor and calcium channel regulator, transcript variant 1
NM_177987	*TUBB8*	2.099369	9.75E-05	Tubulin beta 8 class VIII
NM_173479	*WDR88*	2.107153	0.001438	WD repeat domain 88
NM_153456	*HS6ST3*	2.152067	0.020712	heparan sulfate 6-O-sulfotransferase 3
NM_016445	*PLEK2*	2.234285	0.018735	pleckstrin 2
NM_000589	*IL4*	2.32359	0.016843	interleukin 4, transcript variant 1

NM_001482	*GATM*	2.353803	0.017757	glycine amidinotransferase
NM_004055	*CAPN5*	2.423842	0.01616	calpain 5
NM_000723	*CACNB1*	2.477782	0.028337	calcium voltage-gated channel auxiliary subunit beta 1, transcript variant 1
NM_001206897	*ALDH1A2*	2.516417	0.046497	aldehyde dehydrogenase 1 family member A2, transcript variant 4
NM_001099773	*CYP11A1*	2.518821	0.018445	cytochrome P450 family 11 subfamily A member 1, transcript variant 2
NM_206996	*SPAG17*	2.535975	0.001588	sperm associated antigen 17
NM_005629	*SLC6A8*	2.645453	0.031314	solute carrier family 6 member 8, transcript variant 1
NM_001039966	*GPER1*	2.690041	0.011325	G protein-coupled estrogen receptor 1, transcript variant 3
NM_002188	*IL13*	3.402201	0.022008	interleukin 13
NM_003802	*MYH13*	3.526279	0.001532	myosin heavy chain 13

Table S7

KEGG analysis of signaling pathways among differentially expression transcripts comparing ZFX-silenced CML $CD34^+$ cells with their controls.

**Pathway ID**	**Description**	***P* value**	**Enrich factor**	**Genes**
hsa00053	Ascorbate and aldarate metabolism	6.29E-04	16.79	*ALDH7A1* *UGT2B7*
hsa00140	Steroid hormone biosynthesis	5.88E-03	7.66	*CYP11A1* *UGT2B7*
hsa00260	Glycine, serine and threonine metabolism	1.15E-03	13.64	*ALDH7A1* *GATM*
hsa00330	Arginine and proline metabolism	5.06E-03	8.09	*ALDH7A1* *GATM*
hsa00830	Retinol metabolism	8.10E-03	6.82	*ALDH1A2* *UGT2B7*
hsa00980	Metabolism of xenobiotics by cytochrome P450	1.08E-02	6.15	*UGT2B7* *GSTP1*
hsa00982	Drug metabolism	1.16E-02	5.98	*UGT2B7* *GSTP1*
hsa04310	Wnt signaling pathway	1.43E-02	4.34	*WNT3* *SFRP5* *LEF1*
hsa04660	T cell receptor signaling pathway	3.29E-02	4.04	*ICOS* *IL4*
hsa04664	Fc epsilon RI signaling pathway	1.44E-02	5.53	*IL13* *IL4*
hsa04672	Intestinal immune network for IgA production	3.85E-03	8.91	*ICOS* *IL4*
hsa04916	Melanogenesis	2.77E-02	4.32	*WNT3* *LEF1*
hsa05146	Amoebiasis	3.14E-02	4.12	*SERPINB9* *COL2A1*
hsa05215	Prostate cancer	1.98E-02	4.91	*GSTP1* *LEF1*
hsa05217	Basal cell carcinoma	5.32E-03	7.94	*WNT3* *LEF1*
hsa05310	Asthma	1.05E-03	14.08	*IL13* *IL4*
hsa05412	Arrhythmogenic right ventricular cardiomyopathy (ARVC)	1.21E-02	5.9	*CACNB1* *LEF1*

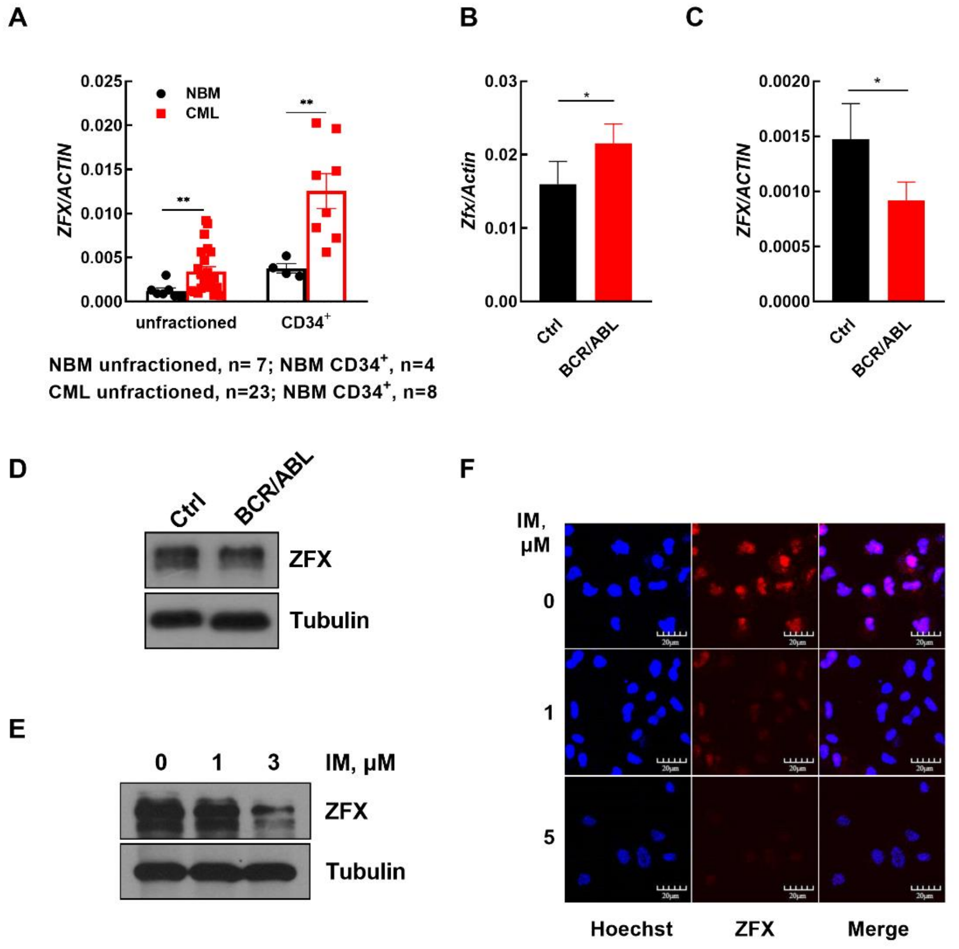

Fig. S1 Zinc finger protein X-linked is upregulated in chronic myeloid leukemia cells and decreases upon imatinib methylate treatment. **A** The expression of *zinc finger protein X-linked* (*ZFX*) in unfractioned BMCs and CD34$^+$ cells from healthy donors and CML patients was assessed with RT-qPCR. **B** The expression of *Zfx* in BaF3 cells upon BCR/ABL transduction was measured by RT-qPCR. **C** The expression of *ZFX* in NBM CD34$^+$ cells upon BCR/ABL overexpression was assessed by RT-qPCR. **D** The expression of Zfx upon BCR/ABL transduction in BaF3 cells was analyzed by Western blotting. **E** K562 cells were treated with various concentrations of imatinib methylate (IM), and ZFX expression was detected by Western Blotting. **F** The expression of ZFX in CD34$^+$ cells from CML patient in chronic phase with or without IM treatment was analyzed by cofocal microscopy. The representative photos are shown. The scale bar is 20 μm. Data are presented as the mean ± SEM, and Student's *t test* was used to estimate the *P* values (**P* < 0.05 and ***P* < 0.01)..

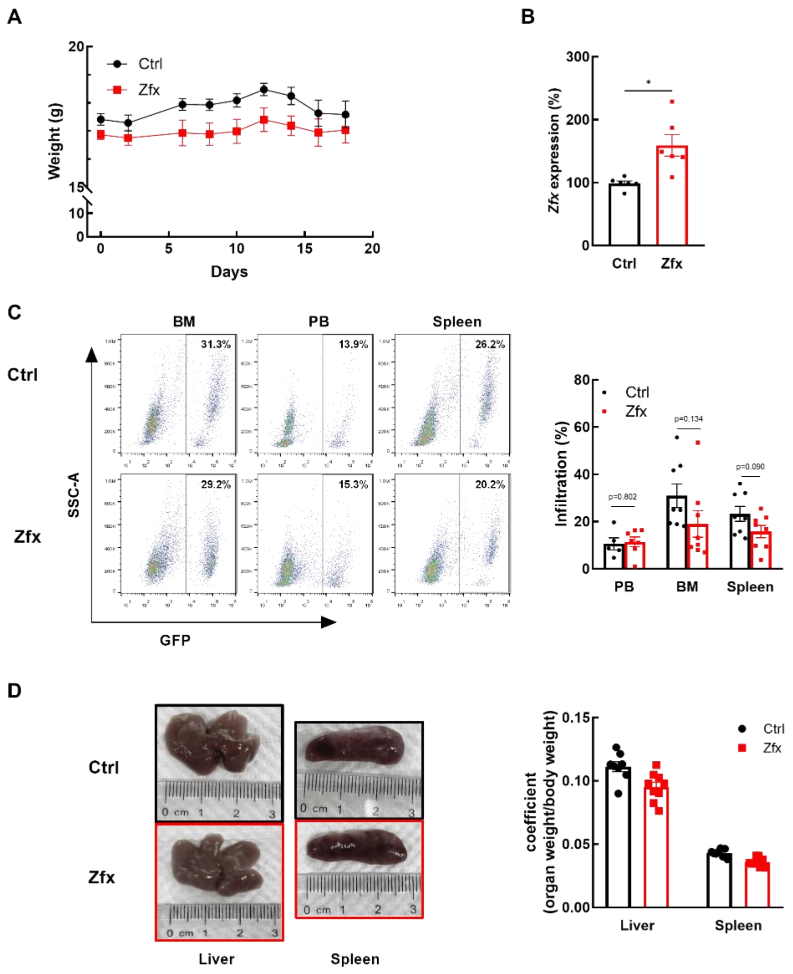

Fig. S2 Zfx overexpression promotes leukemia generation induced by BaF3-BCR/ABL cells.

A The weight of the control (Ctrl) and Zfx-overexpressing groups of mice was monitored after tail vein injection. **B** The diseased mice from the control and Zfx-overexpressing groups were dissected, and leukemic cells were collected and subjected to RT-qPCR analysis for *Zfx* expression. **C** The cells from the bone marrow (BM), peripheral blood (PB), and spleen of both the control and Zfx-overexpressing group were analyzed by flow cytomety to detect the infiltration of BaF3-BCR/ABL cells. The representative flow cytometry profiles are displayed (left panel) and the infiltration of BaF3-BCR/ABL cells were statistically analyzed (right panel). **D** The typical photos of the liver and spleen of the diseased mice from both groups are displayed (left panel). The coefficients of the liver and spleen of both groups were calculated and compared (right panel). Data are presented as the mean ± SEM, and Student's *t test* was used to estimate the *P* values ($^*P < 0.05$).

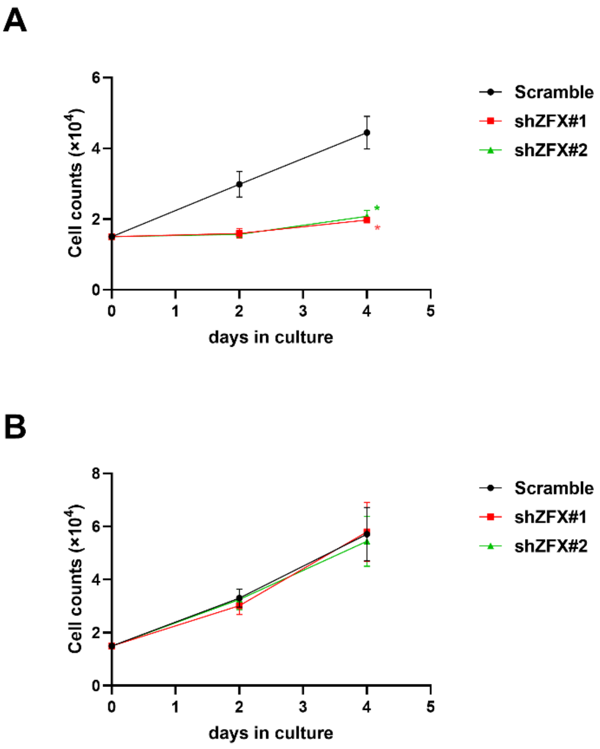

Fig. S3 ZFX silencing specifically inhibits chronic myeloid leukemia CD34$^+$ cells but not normal bone marrow CD34$^+$ cells in liquid culture.

A & B Two independent shRNA sequences against *ZFX* were delivered into chronic myeloid leukemia (CML) CD34$^+$ cells (A, n=4) or normal bone marrow (NBM) CD34$^+$ cells (B, n=4) with lentiviral vectors. FACS purified cells were subjected to proliferation assay. In this assay serum-free media (SFM) were used, which contained a cocktail of cytokines including Flt-3 L (Flt-3 Ligand, 100 ng/mL), SCF (Stem Cell Factor, 100 ng/mL), IL-6 (20 ng/mL), IL-3 (20 ng/mL), and G-CSF (20 ng/mL). Data are presented as the mean ± SEM, and Student's *t test* was used to estimate the *P* values (*P < 0.05).

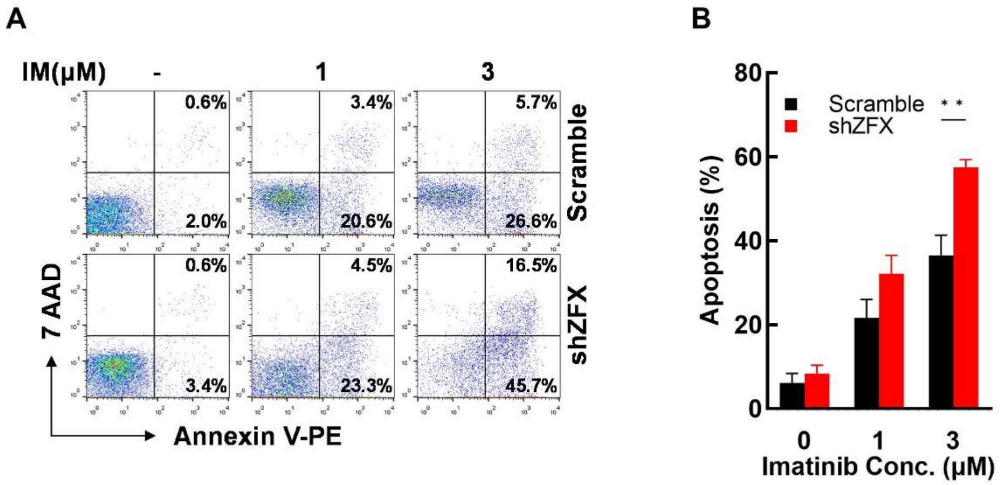

Fig. S4 ZFX silencing promtes Imatinib mesylate induced cell death of K562 cells. The control (scramble) and ZFX-silenced (shZFX) K562 cells were treated with or without imatinib mesylate (IM) and analyzed by Annexin V/7-AAD staining. **A** The representative flow cytomety profiles are displayed. **B** The percentage of apoptotic cells (Annexin V^{+}) were statistically summarized. Data are presented as the mean ± SEM, and Student's *t test* was used to estimate the *P* values (***P* < 0.01).

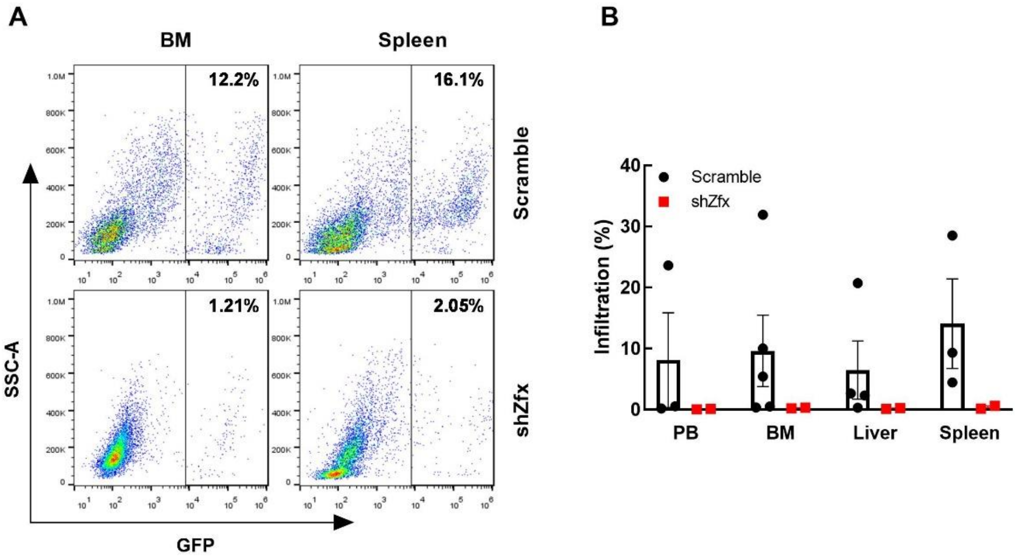

Fig. S5 Zfx silencing decreases the infiltration of BaF3-BCR/ABL cells. Zfx-difcient and control BaF3-BCR/ABL cells were injected intravenously into the irradiated mice.When the mice were diseased and near death, they were sacrificed and dissected to analyze BaF3-BCR/ABL cell infiltration in bone marrow (BM), peripheral blood (PB), spleen, and liver by flow cytomety. **A** The representative flow cytometry profiles to analyze the bone marrow and spleen are displayed. **B** The infiltration of Baf3-BCR/ABL cells were statistically analyzed. Data are presented as the mean ±SEM, and Student's *t test* was used to estimate the *P* values.

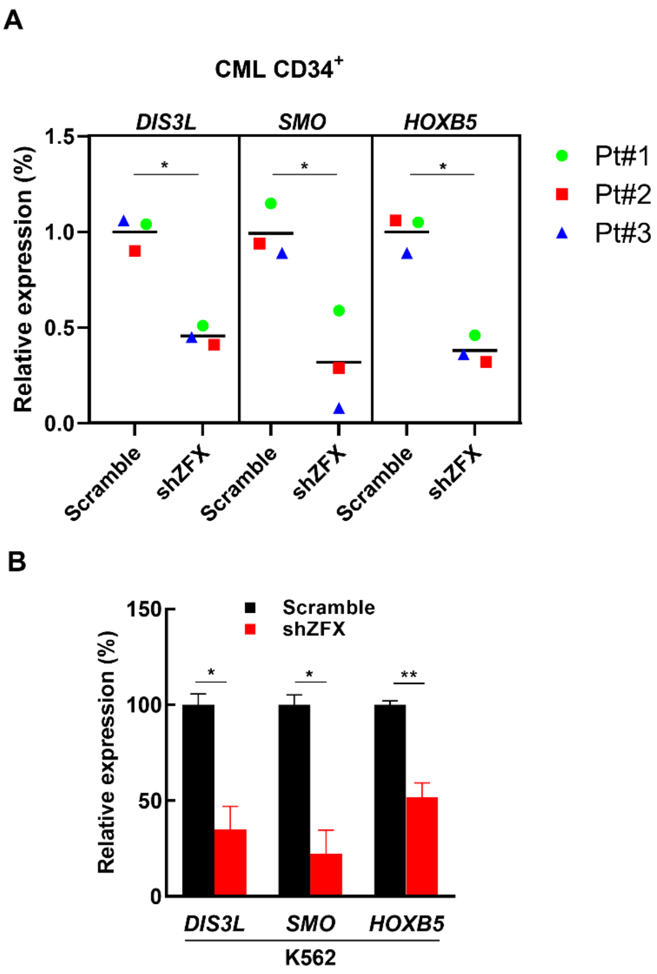

Fig. S6 The expression of *DIS3L*, *SMO*, and *HOXB5* in ZFX-silenced and control CML cells. **A, B** The expression of *DIS3L*, *SMO*, and *HOXB5* in ZFX-silenced and control (scramble) CML CD34$^+$ cells (n=3) and K562 cells were assessed with RT-qPCR (n=3). Data are presented as the mean ± SEM, and Student's *t test* was used to estimate the *P* values (*$P < 0.05$ and ** $P < 0.01$).

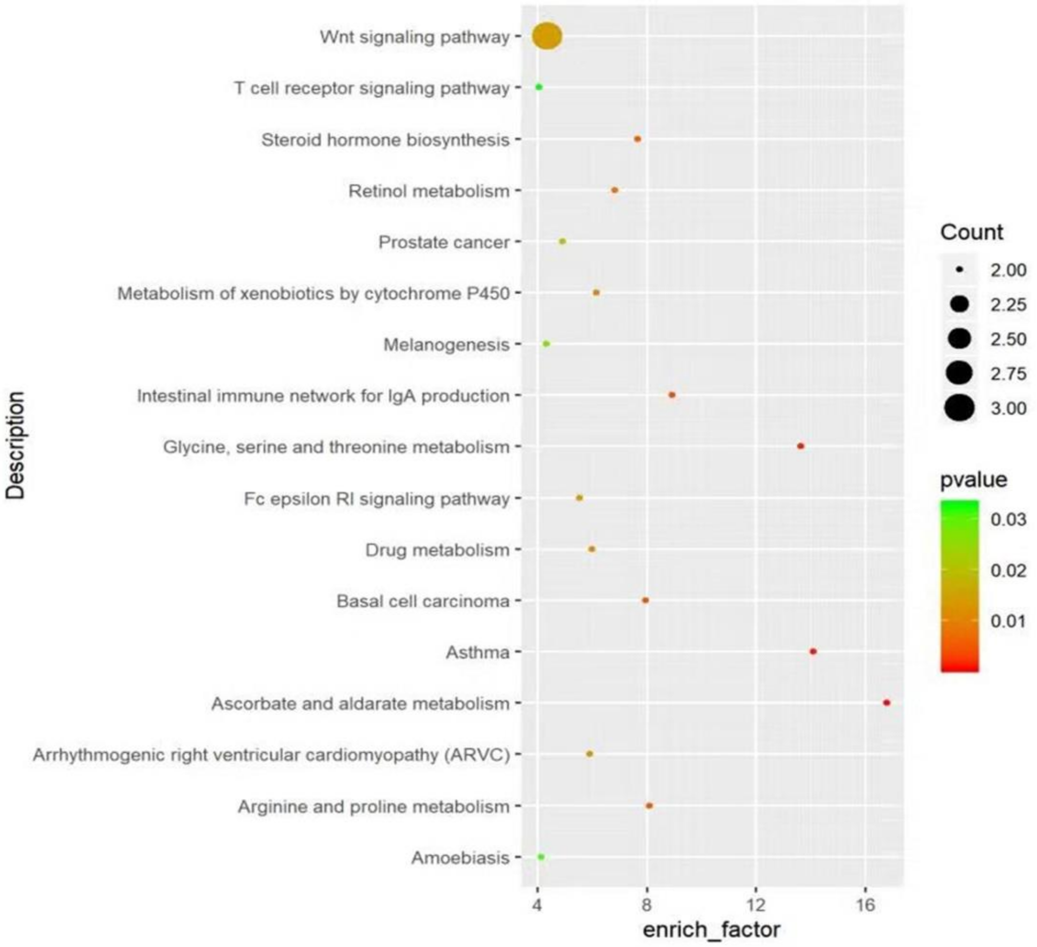

Fig. S7 The KEGG analysis of the differentially expressed transcripts comparing ZFX-silenced with control CML $CD34^+$ cells.

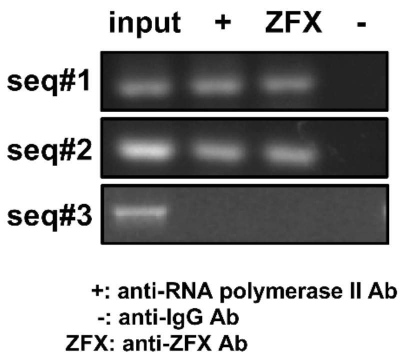

Fig. S8 Chromatin immunoprecipitation (ChIP) was performed to analyze the interaction between ZFX protein and the *WNT3* gene.

A

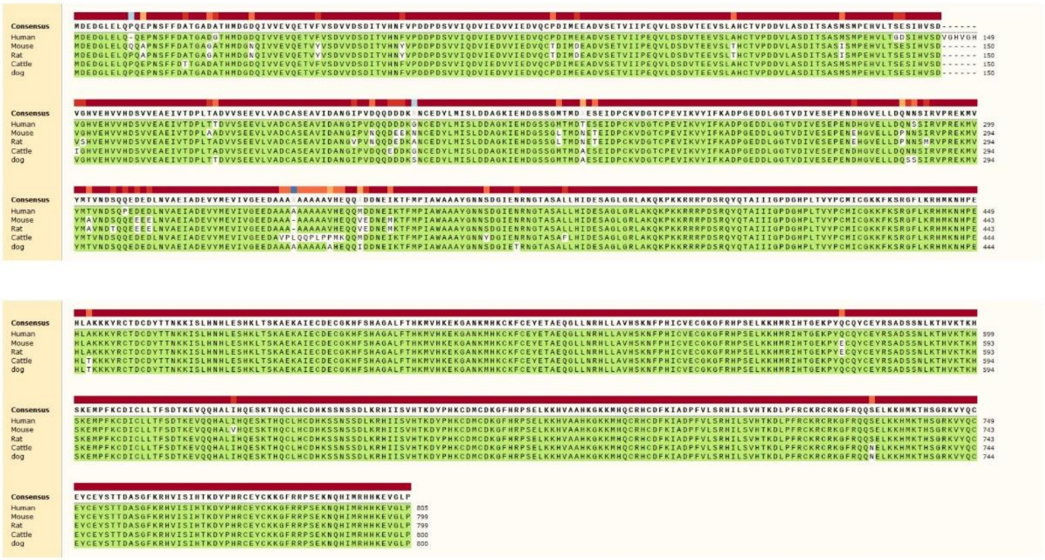

B

Species	Sequence ID	Amino Acids	Molecular mass(kD)	Similarity with human(%)
Human	XP_007529716.1	355	39.65	-
Mouse	NP_001099185.1	355	39.66	99（353/355）

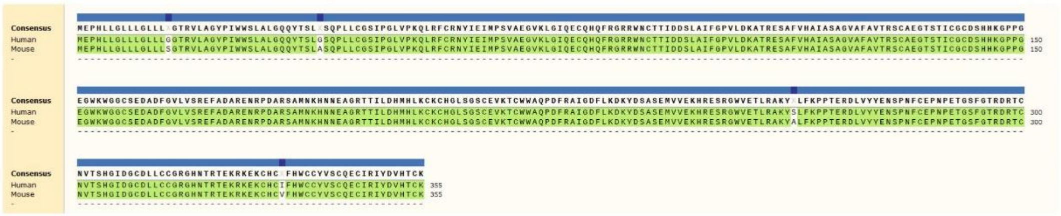

Fig. S9 The alignment analysis of ZFX and WNT3 proteins in mammals. **A** The alignment analysis of ZFX protiens in Human, Mouse, Rat, Cattle, and dog. **B** The alignment analysis of WNT3 proteins betwenn Human and Mouse is shown (lower panel) and the similarity between Human WNT3 and Mouse Wnt3 is summarized (upper panel).

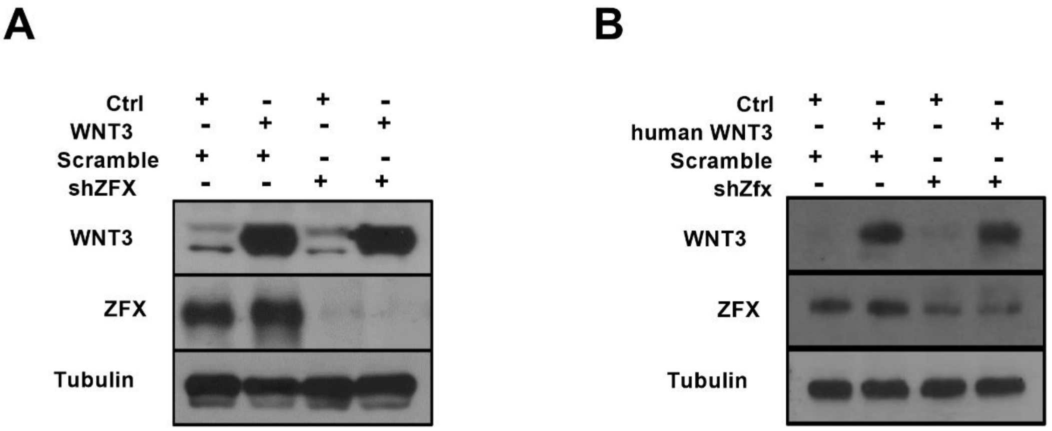

Fig. S10 The expression of WNT3 and ZFX in variously transduced K562 and BaF3-BCR/ABL cells. **A, B** WNT3 and the empty control (Ctrl) were delivered into the control (Scramble) and ZFX-silenced K562 cells (A) and BaF3-BCR/ABL cells (B), the expression of WNT3 and ZFX was analyzed by Western blotting.

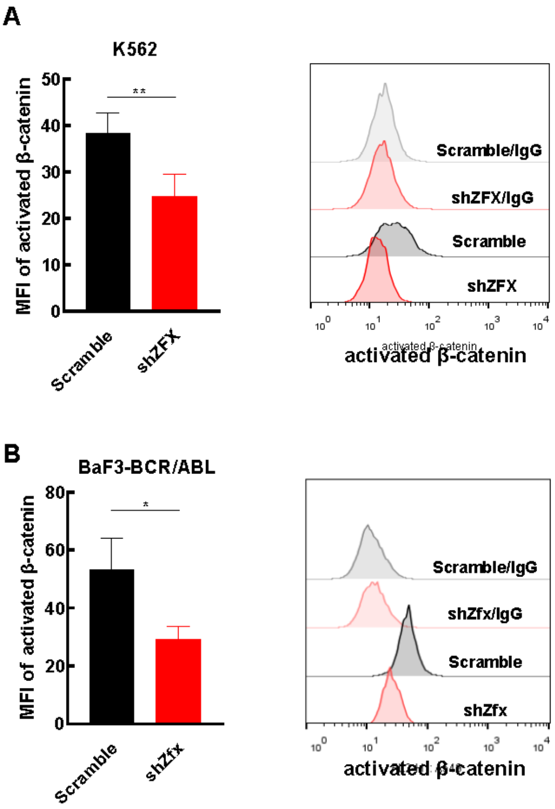

Fig. S11 Activated β-catenin was significant reduced upon ZFX silencing in both K562 and BaF3-BCR/ABL cells. **A,B** The expression of activated β-catenin in K562 cells (A) and BaF3-BCR/ABL cells (B) was detected by flow cytometric analysis and re-analyzed by FlowJo software. Data are presented as the mean ± SEM, and Student's *t test* was used to estimate the *P* values (**P* < 0.05 and ***P* < 0.01). MFI, mean fluorescence intensity.

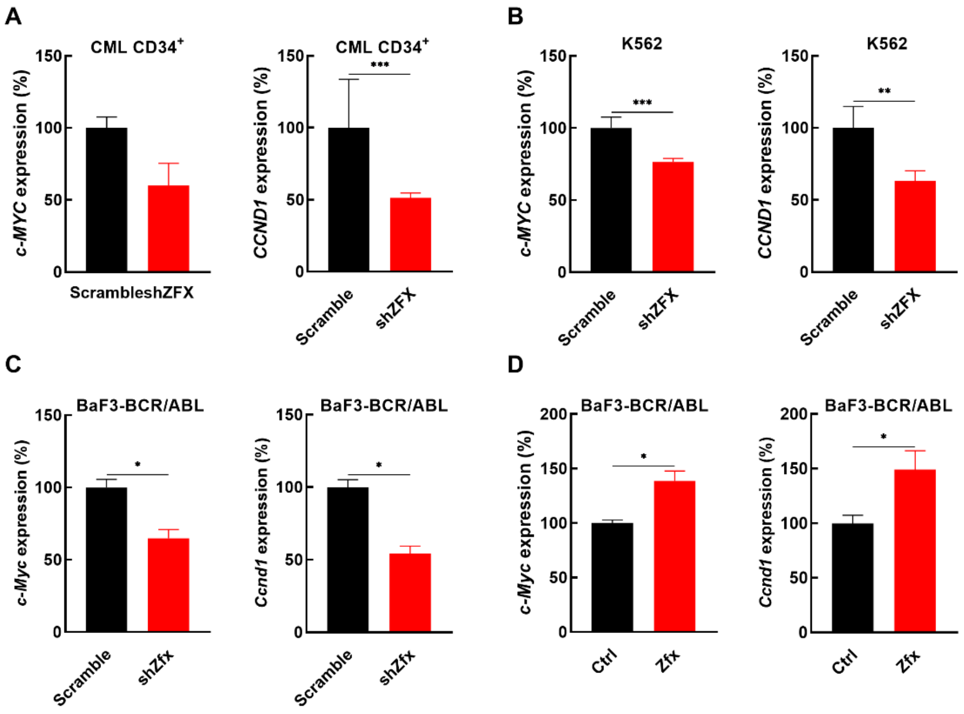

Fig. S12 The expression of *c-MYC* and *CCND1* is regulated by ZFX in various cellular models. **A, B** The expression of *c-MYC* and *CCND1* was measued in ZFX-silenced (shZFX) and control (scramble) CML $CD34^+$ and K562 cells. **C** The expression of *c-Myc* and *Ccnd1* was measured in Zfx-silenced and control (Scramble) Baf3-BCR/ABL cells. **D** The expression of *c-Myc* and *Ccnd1* was assessed in Zfx-overexpressing (Zfx) and control (Ctrl) BaF3-BCR/ABL cells. Data are presented as the mean ± SEM, and Student's *t test* was used to estimate the *P* values (*$P < 0.05$, **$P < 0.01$, and *** $P < 0.001$).
